# Integrated Microbiome and Host Transcriptome Profiles Link Parkinson’s Disease to *Blautia* Genus: Evidence From Feces, Blood, and Brain

**DOI:** 10.3389/fmicb.2022.875101

**Published:** 2022-05-26

**Authors:** Xingzhi Guo, Peng Tang, Chen Hou, Li Chong, Xin Zhang, Peng Liu, Li Chen, Yue Liu, Lina Zhang, Rui Li

**Affiliations:** ^1^Department of Geriatric Neurology, Shaanxi Provincial People’s Hospital, Xi’an, China; ^2^Shaanxi Provincial Clinical Research Center for Geriatric Medicine, Xi’an, China; ^3^Institute of Medical Research, Northwestern Polytechnical University, Xi’an, China

**Keywords:** Parkinson’s disease, microbiome, *Blautia*, 16S, feces

## Abstract

A link between the gut microbiome and Parkinson’s disease (PD) has been intensively studied, and more than 100 differential genera were identified across the studies. However, the predominant genera contributing to PD remain poorly understood. Inspired by recent advances showing microbiota distribution in the blood and brain, we, here, comprehensively investigated currently available fecal microbiome data (1,914 samples) to identify significantly altered genera, which were further validated by comparison to the results from microbiome analysis of blood (85 samples) and brain (268 samples). Our data showed that the composition of fecal microbiota was different from that of blood and brain. We found that *Blautia* was the unique genus consistently depleted across feces, blood, and brain samples of PD patients (*P* < 0.05), despite using rigorous criteria to remove contaminants. Moreover, enrichment analyses revealed that host genes correlated with *Blautia* genus abundance were mainly involved in mitochondrial function and energy metabolism, and mapped to neurodegenerative diseases (NDDs) and metabolic diseases. A random forest classifier constructed with fecal microbiota data demonstrated that *Blautia* genus was an important feature contributing to discriminating PD patients from controls [receiver operating characteristic (ROC)-area under curve (AUC) = 0.704, precision-recall curve (PRC)-AUC = 0.787]. Through the integration of microbiome and transcriptome, our study depicted microbial profiles in the feces, blood, and brain of PD patients, and identified *Blautia* genus as a potential genus linked to PD. Further studies are greatly encouraged to determine the role of *Blautia* genus in the pathogenesis of PD.

## Introduction

Parkinson’s disease (PD) is the second most common neurodegenerative disorder characterized by dopaminergic neuron degeneration in substantia nigra (SN) (Surmeier, 2018). Apart from typical motor impairment, many PD patients also experience non-motor symptoms like gastroparesis and constipation (Chaudhuri et al., 2006; Pfeiffer, 2016), which often happen years before developing motor symptoms (Cersosimo and Benarroch, 2012). Over the last decade, numerous metagenomics studies in human and animals have demonstrated that alterations of gut microbiota composition were closely associated with PD pathophysiology (Qian et al., 2018b; Dong et al., 2020; Aho et al., 2021). Short-chain fatty acids (SCFAs) produced by gut microbiota could affect α-synuclein-induced neuronal cell death and neuroinflammation through regulating the activity of G protein-coupled receptors (GPCRs) (Dalile et al., 2019). Thus, gut microbiota is hypothesized to be a putative diagnostic marker and therapeutic target for PD. Although over 100 genera were reported to be significantly changed in PD patients in two latest meta-analysis (Romano et al., 2021; Shen et al., 2021), no consensus was achieved yet.

Besides the gastrointestinal tract, recent studies have indicated that low-abundance microbiota was also observed in other organs or tissues like tumors, and even in samples from blood, liver and lung, which might serve as a part of microenvironment *in situ* (Lelouvier et al., 2016; Castillo et al., 2019; Klann et al., 2020; Poore et al., 2020; Dohlman et al., 2021). It was reported that age-related changes in intestinal permeability and immune function facilitated the entry of gut bacteria into blood (Nagpal et al., 2018). Indeed, gut permeability was found to be increased (leaky gut) in PD patients (Forsyth et al., 2011). A recent 16S rRNA amplicon study demonstrated the existence of microbiota in the blood of PD patients (Qian et al., 2018a), showing a significant difference in blood microbiota composition between PD patients and controls.

Studies have also evinced the existence of microbiota in the brain of both PD or Alzheimer’s disease (AD) patients and healthy controls, termed as “pathogenic microbial infections” and “brain microbiome,” respectively (Emery et al., 2017; Pisa et al., 2020). As gut microbiota could enter blood owing to an increased gut permeability, microbiota in the brain might be derived from blood, especially in PD patients with blood-brain barrier (BBB) leakage (Emery et al., 2017). Taken together, these findings suggested that microbiota in both blood and brain may play a potential role in the pathogenesis of PD. However, little is known about the microbiota profile in the blood and brain of PD patients, nor 16S rRNA or metagenomics study targeting brain microbiota is now available for PD patients. A recently developed algorithm and software, Kraken2, has been applied in identifying resident microbiota in tumor tissue and blood using RNA-Seq data, with high sensitivity and specificity (Wood et al., 2019; Poore et al., 2020). Thus, it is possible for us to evaluate the microbiota composition in the brain of PD patients using publicly available RNA-Seq data from PD patients’ brain.

In this study, we investigated microbiota landscape among feces, blood and brain samples from PD patients and controls. Meanwhile, microbiota genera with consistent changes across three sample types were selected as candidate “key genera.” To further evaluate the association of “key genera” with PD, a correlation analysis was performed between “key genera” and differentially expressed genes (DEGs), both of which were identified from the same brain RNA-Seq data. In addition, an enrichment analysis was also conducted to estimate the function of DEGs tightly correlated with “key genera.” Moreover, a random forest classifier constructed with microbiota genera data was built to assess the importance of “key genera” in distinguishing PD patients from controls.

## Materials and Methods

### Data Collection and Description

**FIGURE 1:**
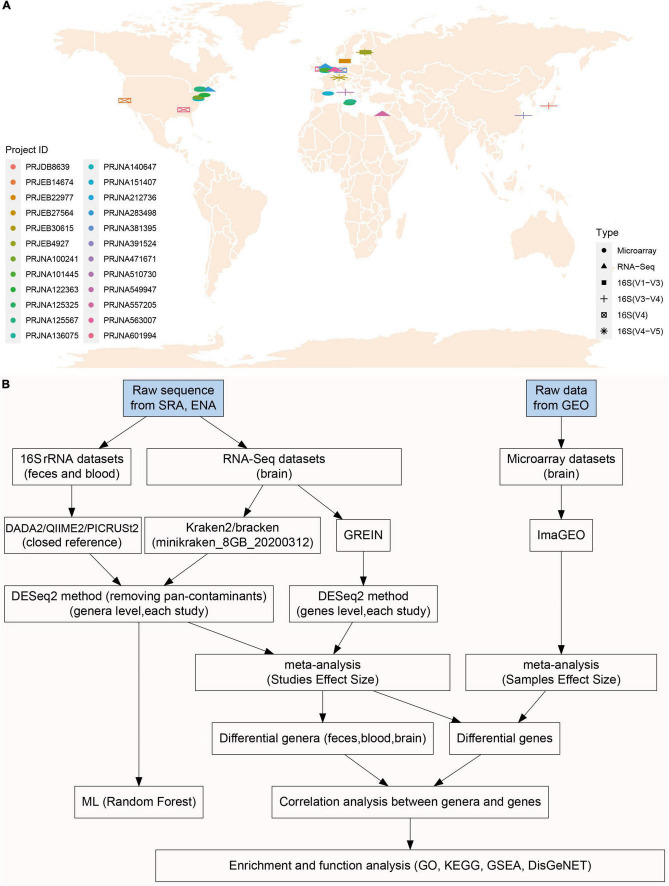
The geographical distribution of included studies and integrative meta-analysis pipeline. There were ten, one, and five studies included in microbiota analysis for feces (16S rRNA), blood (16S rRNA), brain (RNA-Seq) individually, and nine brain microarray studies were enrolled in transcriptome analysis **(A)**. Raw sequence data were downloaded from SRA and ENA database, and brain transcriptome data (microarray) were downloaded from GEO. 16S rRNA sequences were processed with DADA2/QIIME2 pipeline to obtain taxonomy data, and also were processed PICRUSt2 for functional prediction **(B)**. The five RNA-Seq studies used in microbiota analysis (Kraken2) were also used in brain transcriptome analysis (GREIN web platform) **(B)**. Different genera and genes (RNS-Seq) were first calculated with DESeq2 in each study, and then pooled together using fixed effect model meta-analysis. Enrichment analyses (GO, KEGG, and GSEA) were performed using genes significantly associated with commonly changed genera **(B)**.

A comprehensive search was conducted on the Sequence Read Archive (SRA) database^1^ and European Nucleotide Archive (ENA) database^2^ using the following keywords: “Parkinson’s disease,” “blood,” “brain,” “gut,” “feces,” “16S,” “rRNA,” “RNA-Seq,” “transcriptome,” “microbiota,” and “microbiome.” Gene expression microarray data of the brain in PD patients were retrieved in the Gene Expression Omnibus (GEO) database. Only samples from Homo sapiens were included in this study. All metadata sets were downloaded from the SRA database or GEO database according to the unique Project ID of each study. The geographic distribution of all included studies (Figure 1A) and workflow (Figure 1B) for this study was presented in Figure 1.

### Data Processing

The 16S rRNA and RNA-Seq data downloaded from the ENA were processed with QIIME2 2020.8.0 (Bolyen et al., 2019) and Kraken 2.1.1 (Wood et al., 2019) according to the standardized pipeline, respectively. Briefly, the quality of each sequence read was checked by trim-galore (V.0.6.6), and a base with Phred score less than 20 was removed from further analysis. For each 16S rRNA dataset, all reads were input into QIIME2 as demultiplexed sequences, which were then denoised to generate amplicon sequence variants (ASVs) using DADA2 (Callahan et al., 2016). The cluster-features-closed-reference method was used to pick up OTUs. The classify-consensus-vsearch method was applied to assign taxonomy using 99% Greengenes database 13.8 and 99% SILlVA 138 as reference. Recent Shotgun metagenomic data (PRJNA433459) (Qian et al., 2020), which were re-analyzed with Kraken2 in this study, were further used to verify the results obtained from 16S rRNA. For each RNA-Seq dataset, all reads were classified with Kraken2 using minikraken_8GB_20200312 database as reference, and Bracken was then applied to count the relative abundance of taxonomy based on the read length (Wood et al., 2019).

The same RNA-Seq data used above were also applied to detect gene expression levels in the brain. RNA-Seq and microarray data were processed with two web tools, GREIN^3^ (Al Mahi et al., 2019) and ImaGEO^4^ (Toro-Domínguez et al., 2019), respectively. Briefly, unique GSE ID obtained from the GEO was used as query in GREIN and ImaGEO individually, and data were processed with the default parameters. A meta-analysis was conducted automatically to identify DEGs in the ImaGEO after microarray data were processed. All statistical analyses were conducted with R (version 4.0.4) (Chambers, 2008) unless otherwise stated, and P value less than 0.05 was considered statistically significant.

### Characterization of Microbial Communities in Parkinson’s Disease

To alleviate batch effects (hypervariable region, sequencing platform, and country, etc.), microbiota analysis was performed at the genus level. To exclude the impacts of bacterial contamination, those genera previously reported as potential contaminants or widely distributed in the tubes and agents (Supplementary Data 1) (Salter et al., 2014; Davis et al., 2018; Eisenhofer et al., 2019) were removed. To identify contaminants in blank tubes and agents, Salter et al. routinely process the a negative ‘blank’ controls without adding sample template alongside human-derived samples by 16S rRNA and shotgun metagenomics (Salter et al., 2014). A list of contaminant genera was detected in sequenced negative ‘blank’ controls (Supplementary Data 1), which were removed from microbiota analysis in our study. In addition, as gut microbiota is the main source of bacteria in human, we also excluded genera (“pan-contaminants”) exclusively found in the blood and brain. In this study, all data analyses were conducted using the microbiota genera data after removing pan-contaminants unless otherwise stated. The relative abundance of bacteria at the Phylum level was calculated and plotted using the tax_stackplot within amplicon (V.1.1.5) package (Liu Y.-X. et al., 2021). A heatmap of genera with a relative abundance equal or more than 0.5% was also plotted for each sample types (feces, blood, and brain) using the pheatmap (V.1.0.12) package (Kolde and Kolde, 2015). Since the sequencing depth and genera abundance among feces, blood, and brain samples was different, rarefied genera data were only used for diversity analysis. For the α-diversity analysis in the feces and blood or brain (RNA-Seq), 3,000, and 1,000 counts were extracted randomly from each sample without replacement to estimate Observed, Chao1, Shannon, and Simpson evenness indices using the phyloseq (V.1.34.0) package (McMurdie and Holmes, 2013). For β-diversity analysis, the relative abundance of genera profiles were used to calculate β-diversity with the following parameters (dist = “bray,” method = “PCoA,” Micromet = “Adonis”) in amplicon package (Liu Y.-X. et al., 2021).

### Predicted Microbiota Function

To assess the difference in microbiota function between PD patients and controls, we used Phylogenetic Investigation of Communities by Reconstruction of Unobserved States (PICRUSt2) to determine the predicted function of gut microbial communities from 16S rRNA sequencing data (Douglas et al., 2020). The KEGG Orthology (KO) metagenome prediction data were obtained using the picrust2_pipeline.py script in PICRUSt2. After that, the predicted KO abundances data were categorized into KEGG pathway abundances of Level 2 and Level 3. Finally, a meta-analysis was performed to identify the different KEGG pathways of Level 2 and Level 3 between PD patients and controls.

### Meta-Analysis of Genera and Gene Level

The genus-level abundance data were processed with qiime2R (V.0.99.4) and phyloseq package (Bisanz, 2018). Since pooling summary statistics from each study was more robust in alleviating between-study heterogeneity than pooling raw data directly from all studies (Duvallet et al., 2017), the genus-level abundance data from each study were analyzed with DESeq2 (Love et al., 2014). The summary statistics (log2 fold-changes and 95%CIs) of a single genus from each study were then pooled with a fixed-effect meta-analysis using the meta (V.4.18-2) package (Schwarzer and Schwarzer, 2012). Due to only one study available for blood 16S rRNA, no meta-analysis was performed. The significantly different genera between PD patients and controls across brain, blood, and feces samples were summarized to obtain the common genera/genus with the same change direction, and a corresponding heatmap was plotted using the ggplot2 (V.3.3.3) package (Wickham, 2011).

For gene expression data with RNA-Seq (GREIN processed), raw counts data from each study were used to calculate log2 fold-changes of each gene using the DESeq2 (V.1.30.1) package (Love et al., 2014). After that, a meta-analysis was performed to identify the DEGs between PD patients and controls using the meta package (Schwarzer and Schwarzer, 2012).

### Correlation Analysis and Enrichment Analysis

To explore the potential interaction between *Blautia* genus and host genes, a correlation analysis was performed between the *Blautia* genus and each host gene (RNA-Seq) using the corrplot (V.0.89) package with Spearman method (Wei et al., 2017). Correlation coefficients with a magnitude above 0.3 were considered to have a correlation.

Host genes associated with *Blautia* genus (*r* > 0.3) were enrolled in enrichment analyses, including Gene Ontology (GO) (Gene Ontology Consortium, 2004), Kyoto Encyclopedia of Genes and Genomes (KEGG) (Kanehisa and Goto, 2000), and Gene Set Enrichment Analysis (GSEA) (Subramanian et al., 2005) using the clusterProfiler (V.3.18.1) (Yu et al., 2012) and enrichplot (V.1.10.2) package (Yu, 2018). Briefly, the gene symbols with combined effect sizes were input as a query gene list and a *P* value cutoff was set to 0.05. Meanwhile, further enrichment analyses were also conducted based on DEGs (RNA-Seq or RNA-Seq and Microarray) correlated to *Blautia* genus. Moreover, to explore the relationship between *Blautia-*associated genes and diseases category (Piñero et al., 2016), *Blautia-*associated DEGs (overlapped between RNA-Seq and microarray) were also used to conduct diseases enrichment analysis with the disgenet2r (V.0.99.2) package in DisGeNET database (Gutiérrez-Sacristán et al., 2016).

### Model Construction, Genera Extraction, and Model Evaluation

To verify the importance of *Blautia* genus in discriminating PD patients from controls, we used the microbiota data to build a random forest (RF) model with stratified 5-fold cross-validation (CV) using the SIAMCAT (V.1.10.0) package (Wirbel et al., 2021). The features used for model building consist of patients’ metadata as well as genera with a relative abundance greater than 0.1% across all the samples. The metadata features consisted of age, gender, body mass index (BMI), or hypervariable region (16S rRNA). The RF models were built with 1,000 estimator trees and default parameters to select the proportion of features for each tree. The stratified 5-fold cross-validation was executed to configure training and testing data sets. The top 30 features from the top-performing model were selected as “important features.” Finally, the receiver operating characteristic curve (ROC) and precision-recall curve (PRC) with area under curve (AUC) was used to assess the performance of models.

## Results

### Different Taxonomic Composition and Diversity of the Feces, Blood, and Brain

**FIGURE 2:**
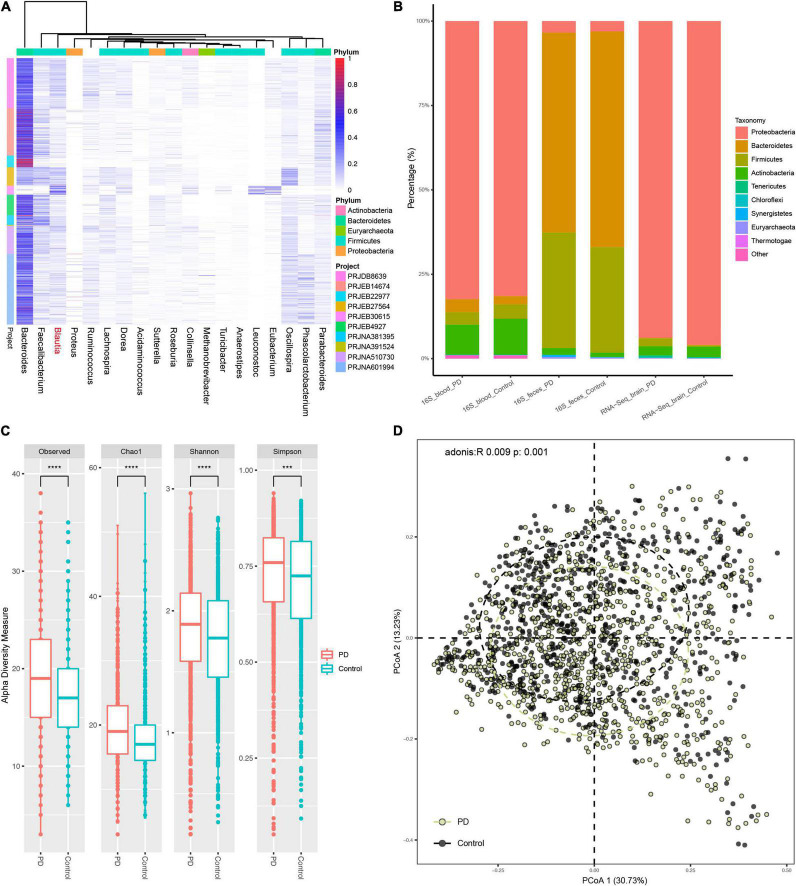
The fecal microbiota composition in PD patients and controls. All microbiota composition data here were first filtered with the pan-contaminants list before further analysis. There were 18 genera with an average relative abundance more than 0.5% across all fecal samples **(A)**. **(B)** Showed the microbiota composition among feces, blood, and brain samples of patients with PD and controls at the genus level after removing the contaminants (not pan-contaminants). The major fecal Phylum were *Firmicutes* and *Bacteroidetes*, while the main Phylum in blood and brain were *Proteobacteria* and *Actinobacteria* **(B)**. There was obvious *Firmicutes* proportion in PD brain, which were almost depleted after filtering the brain genera data using pan-contaminants list (Supplementary Figure 2). The α-diversity, including Observed, Shannon, Simpson and Chao1 indices, was significantly increased in the feces of PD patients compared to controls **(C)**. There was an obvious difference in β-diversity between PD patients and controls with Bray–Curtis distance (adonis: *R* = 0.009, *P* = 0.001) **(D)**. ****: *P* value less than 0.0001, ***: *P* value less than 0.005.

A total of 16 studies, including ten for feces, one for blood, and five for the brain, with 2,883 participants were included in this study before data cleaning. All the Project IDs and details of metadata were provided in Table 1 (Supplementary Table 1 and Supplementary Data 2). Genera reported as potential contaminants and non-detectable in fecal microbiome were termed as “pan-contaminants” and were removed before microbiota analysis. For the feces, 311 genera were identified across 2,252 individual samples. After removing contaminants (Supplementary Data 3), 197 genera retained and 28 genera had an average relative abundance greater than 0.5% across all samples (Figure 2A). For the blood, 328 genera were identified across 86 individual samples with 100 retained after excluding pan-contaminants (Supplementary Data 4), and 14 genera had an average relative abundance greater than 0.5% across all samples (Supplementary Figure 1A). For the brain, 1,197 genera were identified across 376 individual samples with 116 retained after removing pan-contaminants (Supplementary Data 5), and 18 genera had an average relative abundance greater than 0.5% across all samples (Supplementary Figure 1B). For fecal studies, samples overlapping between PRJEB14674 and PRJNA601994 were de-duplicated, and 1,914 samples were finally included. There were four replicated runs of each sample in PRJNA557205, and the RNA-Seq data from the first run (run accession IDs) of each sample were used in both microbiota analysis and transcriptome analysis (Li et al., 2020).

**TABLE 1:** Summary of characteristics of the included projects for microbiome analysis.

Project	Gender	BMI (SD)	Age (SD)	Country	Platform	Type	Source	Control (n)	PD (n)
PRJDB8639	NA	NA	NA	Japan	Illumina MiSeq	16S(V3-V4)	feces	137	223
PRJEB14674^#^	Female (43.7%)	27.2 (5.56)	67.4 (3.96)	United States	Illumina MiSeq	16S(V4)	feces	135	213
PRJEB22977	NA	NA	NA	Denmark	Illumina MiSeq	16S(V1-V3)	feces	32	52
PRJEB27564	NA	NA	NA	Finland	Illumina MiSeq	16S(V3-V4)	feces	65	68
PRJEB30615	Female (40.6%)	NA	62.2 (17.9)	Germany	Illumina MiSeq	16S(V4-V5)	feces	25	39
PRJEB4927	Female (50.0%)	NA	64.8 (6.21)	Finland	454GS	16S(V1-V3)	feces	74	74
PRJNA283498	Female (0%)	NA	73.0 (12.2)	United States	Illumina HiSeq2000	RNA-Seq	brain	44	29
PRJNA381395	NA	NA	NA	Germany	Illumina MiSeq	16S(V4)	feces	70	40
PRJNA391524	NA	NA	NA	China	Illumina MiSeq	16S(V3-V4)	feces, blood	90	90
PRJNA471671	Female (33.3%)	NA	78.7 (9.11)	Israel	NextSeq 500	RNA-Seq	brain	29	46
PRJNA510730	NA	NA	NA	Italy	Illumina MiSeq	16S(V3-V4)	feces	84	118
PRJNA549947	NA	NA	NA	Israel	NextSeq 500	RNA-Seq	brain	26	43
PRJNA557205*	Female (11.1%)	NA	75.3 (5.10)	Israel	NextSeq 500	RNA-Seq	brain	12	24
PRJNA563007	Female (37.5%)	NA	NA	Netherlands	Illumina HiSeq2000	RNA-Seq	brain	8	8
PRJNA601994^#^	Female (46.8%)	NA	67.9 (8.94)	United States	Illumina MiSeq	16S(V4)	feces	316	524

*NA, not available; BMI, body mass index; SD, standard deviation.*

*Four duplications for each sample.

*^#^The samples’ raw sequence data in PRJEB14674 were overlapped with PRJNA601994, and were removed from PRJNA601994 before data analysis.*

Microbiota composition data without reported contaminants showed that the main flora in feces were *Firmicutes* and *Bacteroidetes*, while major flora in blood and brain were *Proteobacteria*, followed by a small part of *Actinobacteria* (Figure 2B). In fecal samples, patients with PD harbored a higher relative abundance of *Firmicutes*, *Actinobacteria*, and *Synergistetes*, but a lower relative abundance of *Bacteroidetes* (Figure 2B and Supplementary Figure 2A), contributing to an increased ratio of *Firmicutes* to *Bacteroidetes* (F/B) in the feces of patients with PD. The *Firmicutes* phylum in PD brain were almost depleted after excluding the pan-contaminants (Supplementary Figure 2A).

The results of α-diversity analysis revealed that the α-diversity of fecal microbiota was significantly higher in PD patients than controls (Figure 2C), but no significant difference was found in the blood and brain (Supplementary Figures 2B,D). For β-diversity, the results of principal coordinates analysis (PCoA) with Bray–Curtis distance showed that the bacterial communities in the feces differed significantly between PD patients and controls (Figure 2D) (*p* < 0.001, PERMANOVA by Adonis), but not in the blood and brain (Supplementary Figures 2C,F).

### Predicted Function in PICRUSt2

The results of PICRUSt2 predictions showed that top ten altered KEGG pathways of Level 2 were mainly associated with neurodegenerative diseases, infectious diseases, cancer, transport, and catabolism etc. (Supplementary Figure 3A). Data from the top ten altered KEGG pathways of Level 3 showed an increased abundance of betalain biosynthesis, amoebiasis, and hepatitis C, while decreased in beta-lactam resistance, Ubiquitin system, isoquinoline alkaloid biosynthesis, beta-alanine metabolism, etc. (Supplementary Figure 3B). A full list of all the changed KEGG pathways of both Level 2 and Level 3 were presented in Supplementary Tables 2, 3, respectively.

### Different Genera Between Patients With Parkinson’s Disease and Controls in the Feces, Blood, and Brain

**FIGURE 3:**
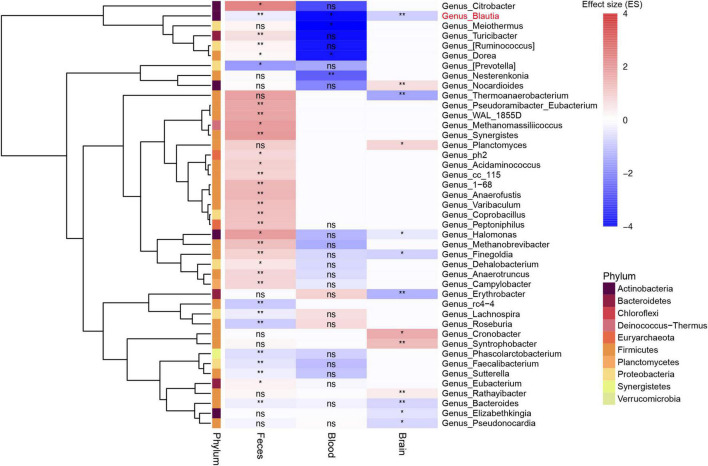
Heatmap showing the different genera among feces, blood, and brain between patients with PD and controls. All microbiota data here were first filtered with the pan-contaminants list. Most of genera in feces and blood were not shown in the brain. *Blautia* was the only genus decreased among feces, blood, and brain samples (labeled in red). *Bacteroides* genus was also found to be decreased in both feces and brain samples, but not in blood samples. The blank rectangle area in the heatmap without any annotation means genera not found in corresponding sample type. **: *P* value less than 0.01, *: *P* value less than 0.05, ns: no significance.

In the feces, the pooled results of taxonomical classification by Greengenes showed that 32 genera were significantly different between PD patients and controls (*P* < 0.05, Supplementary Table 4). Nine of the 32 different genera were depleted, while 23 were enriched in PD. Meanwhile, the pooled results of taxonomical classification by SILVA showed that there were 23 genera significantly altered in PD patients (*P* < 0.05, Supplementary Table 5 and Supplementary Data 6), and 15 of them overlapped with the different genera identified using Greengenes alignment (Supplementary Figure 4). Using shotgun metagenomics data (PRJNA433459), we found that 377 genera were significantly different between PD patients and controls (*P* < 0.05, Supplementary Data 7), with 251 genera depleted and 126 enriched in PD, respectively. In the blood, the results showed that all four different genera were significantly decreased in PD patients compared to controls (*P* < 0.05, Supplementary Table 6). In the brain, a total of 13 genera was found to be significantly different between PD patients and controls (*P* < 0.05, Supplementary Table 7), with 8 genera depleted and 5 genera enriched in PD (Figure 3).

The *Halomonas* and *Finegoldia* genus was depleted in the brain, but enriched in the feces of PD patients (*P* < 0.05) (Figure 3). Meanwhile, the *Dorea* genus was increased in the blood, but decreased in the feces (*P* < 0.05). The *Bacteroides* genus were depleted in the feces and brain of patients with PD (*P* < 0.05) (Figure 3). After comparing the different genera among feces, blood and brain samples, we found that only *Blautia* genus consistently altered (depleted) across all sample types in PD (*P* < 0.05) (Figure 3). Further analysis at species level using gut shotgun metagenomics data showed that *Blautia argi, Blautiacoccoides, Blautia sp. SC05B48, and Blautiahansenii* species were decreased in the feces of patients with PD as compared to controls (Supplementary Table 8).

### Correlation Analysis Between *Blautia* Genus and Host Genes in the Brain

**FIGURE 4:**
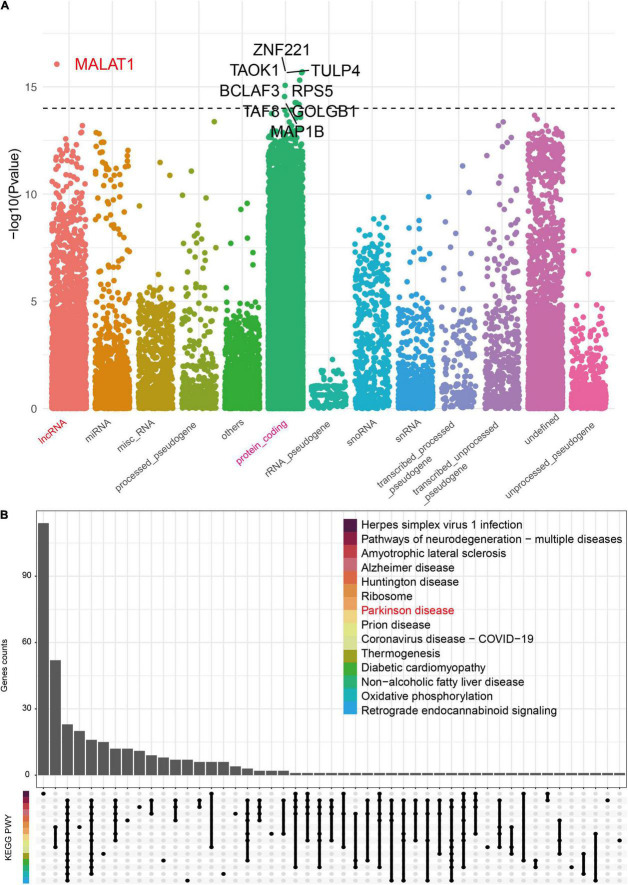
The biotype distribution of genes significantly associated with *Blautia* genus. A Spearman correlation analysis was performed between *Blautia* genus with all host genes identified from RNA-Seq using the GREIN (http://www.ilincs.org/apps/grein/?gse=). The relative abundance of microbiota composition data and log2 transformed genes counts were used to calculate r values with limma (V.3.46.0) package. The absolute *r* values ranged from 0.12 to 479 with P less than 0.05. An absolute *r* value greater than 0.3 and *P* value less than 0.05 was considered as significant correlation. The biotype of all genes was classified with the biomaRt (V.2.46.3) package and was described in Manhattan plot **(A)**. The top nine genes (including lncRNA) with an absolute r value greater than 0.45 were labeled with corresponding gene symbol **(A)**. An UpSet plot showed the KEGG results of *Blautia* genus correlated genes (|r| > 0.3 and *p* < 0.05) **(B)**.

To investigate the potential role of *Blautia* genus in PD, Spearman correlation analyses between *Blautia* genus and brain genes were conducted by integrating the microbiome and host transcriptome data obtained from the same raw RNA-Seq data. The results showed that 17,216 genes were associated with *Blautia* genus (*P* < 0.05), with an absolute *r* value ranging from 0.12 to 0.479. After removing those genes with an absolute *r* value less than 0.3, we found 3,583 genes remained associated with *Blautia* genus, with 1,775 and 1,808 genes having positive and negative relationship with *Blautia* genus (Supplementary Data 8), respectively. Biotype distribution analysis using the biomaRt (V.2.46.3) package showed that, besides protein coding class, there were 78 and 18 genes mapped to lncRNA and miRNA class, respectively (Supplementary Table 9). The top nine genes labeled with gene symbol in Figure 4A had an absolute r value greater than 0.45. Eight of the nine genes belong to protein encoding gene, while only MALAT1 was a lncRNA.

### Enrichment Analysis of *Blautia* Genus Correlated Genes

To assess whether host genes associated with *Blautia* genus were involved in the pathogenesis of PD, we then implemented three functional enrichment analyses, including KEGG, GO, and GSEA. The results of KEGG analysis showed that these genes were mainly related neurodegenerative diseases, including PD, AD, Huntington’s disease (HD), and amyotrophic lateral sclerosis (ALS), and metabolism related diseases, including diabetic cardiomyopathy (DCM) and non-alcoholic fatty liver disease (NAFLD) (Figure 4B). Data from GO enrichment analysis showed that these genes were mainly mapped to proteins transportation, immune response, energy metabolism, and mitochondrial function (Supplementary Figure 5). Meanwhile, the above results were further confirmed by GSEA-based KEGG and GO enrichment analysis (Supplementary Figures 6A,B).

### Enrichment Analysis of *Blautia* Genus Associated Differentially Expressed Genes in Parkinson’s Disease

**FIGURE 5:**
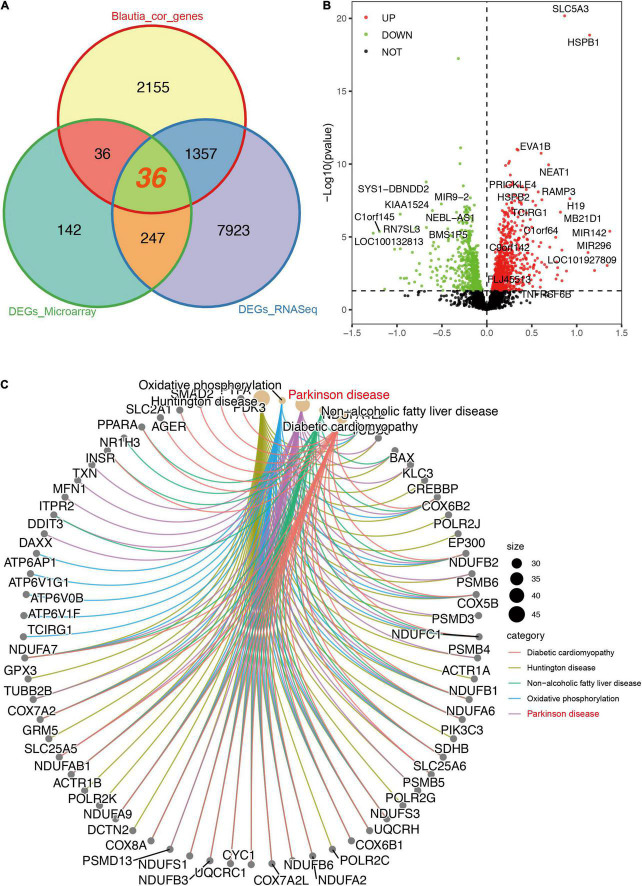
Descriptions of DEGs (RNA-Seq) significantly associated with *Blautia* genus. A Venn graph showing the intersected DEGs between RNA-Seq and microarray data, and genes significantly correlated with *Blautia* genus (|r| > 0.3 and *p* < 0.05). A total of 1,357 DEGs associated with *Blautia* genus were only detectable in RNA-Seq data, but not in microarray data **(A)**. On the other hand, 36 DEGs associated with *Blautia* genus were found in both RNA-Seq and microarray data **(A)**. Over 98% genes identified in microarray data were also detectable in RNA-Seq data (Supplementary Figure 7A). A volcano plot showed the distribution of *Blautia* genus associated genes identified from RNA-Seq data **(B)**. Red dots indicated increased expression, while green dots indicated decreased expression in PD. Black dot signified no statistical significance **(B)**. Those DEGs with absolute effect size greater than 0.5 were labeled with corresponding gene symbol. The CNet plot showed the KEGG results of DEGs significantly associated with *Blautia* genus **(C)**. The top five enriched KEGG terms mapped by the DEGs were DCM, PD (labeled with red), HD, NAFLD, and oxidative phosphorylation **(C)**.

To identify the DEGs in the brain of PD patients, a meta-analysis was performed using RNA-Seq and microarray data, respectively. Using brain RNA-Seq data, we found that 9,563 DEGs were significantly different between PD patients and controls (Supplementary Data 9), with 4,618 decreased genes and 4,945 increased genes, respectively. A total of 1,393 DEGs were significantly associated with *Blautia* genus (*r* > 0.3 and *P* < 0.05) (Figure 5A), with 569 downregulated genes and 824 upregulated genes (Figure 5B) individually. Among the 1,393 DEGs, 759 DEGs were positively correlated with *Blautia* genus, while 634 DEGs were negatively associated with *Blautia* genus (Supplementary Data 10).

Using brain microarray data, a total of 3,237 genes were detected in microarray and over 98% of the genes were also identified in RNA-Seq (Supplementary Figure 7). Only 67 genes were found exclusively in microarray, but 32,474 gene transcripts identified in RNA-Seq were not covered in microarray (Supplementary Figure 7A). There were 460 DEGs significantly changed in patients with PD using microarray data (Supplementary Data 11). Finally, only 36 DEGs correlated to *Blautia* genus were found in both RNA-Seq and microarray data (Figure 5A), and 26 out of the 36 genes were negatively associated with *Blautia* genus (Supplementary Figure 8), respectively.

**FIGURE 6:**
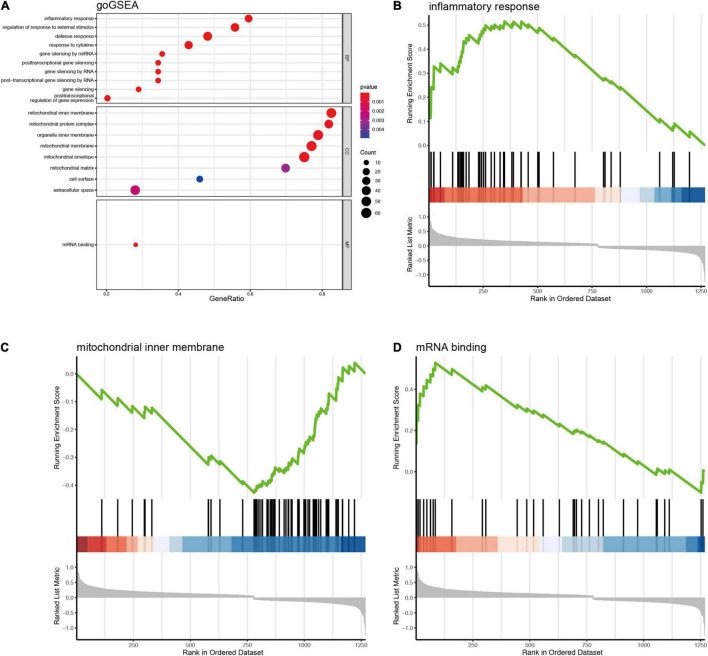
GSEA based GO enrichment analysis of DEGs (RNA-Seq) significantly associated with *Blautia* genus. The gseGO showed that DEGs associated with *Blautia* genus (|r| > 0.3 and *p* < 0.05) were mainly targeted to inflammatory response and mitochondrial function **(A)**. *Blautia* genus related DEGs enriched in inflammation were significantly increased in PD **(B)**. Most of *Blautia* genus related DEGs involved in mitochondrial function were decreased **(C)**. **(D)** Showed that the DEGs associated with mRNA binding were increased.

Since 1,357 DEGs associated *Blautia* genus were exclusively identified in RNA-Seq data, but not covered in microarray data, thus, in order to avoid losing potential important information, we first performed an enrichment analysis based on RNA-Seq derived DEGs. The results of GO enrichment analysis showed that the 1,393 DEGs associated *Blautia* genus were mainly located in mitochondrial membrane, participating in regulating energy metabolism and mitochondrial function (Supplementary Figure 7B and Supplementary Data 12). Data from KEGG analysis demonstrated that the 1,393 DEGs were mainly mapped to PD, HD, DCM, and NAFLD (Figure 5C and Supplementary Data 13), which was further confirmed by GSEA-based KEGG analysis (Supplementary Figures 9A–D and Supplementary Data 14). Meanwhile, GSEA-based GO analysis showed that these DEGs were mainly targeted in inflammatory response (Figures 6A,B), mitochondrial function (Figure 6C), and miRNA binding (Figure 6D and Supplementary Data 15).

Moreover, we also conducted a GO analysis using the 36 DEGs overlapping between RNA-Seq data and microarray data. The result showed that the 36 DEGs were mainly mapped to pH regulation, protein degradation, and mitochondrial function (Supplementary Figure 10). Several of the 36 DEGs, including KCNJ6, ACOX1, USP9X, SMAD2, SLC8A1, PIK3C3, PDK1, have been previously reported to be implicated in the pathogenesis of PD (Rott et al., 2011; di Val Cervo et al., 2017; Muñoz et al., 2020). Disease enrichment analysis performed in Disgenet2 database showed that the 36 DEGs were mainly associated with nervous system diseases and digestive system diseases (Supplementary Figure 11).

### Microbial Classification Models for Parkinson’s Disease

**FIGURE 7:**
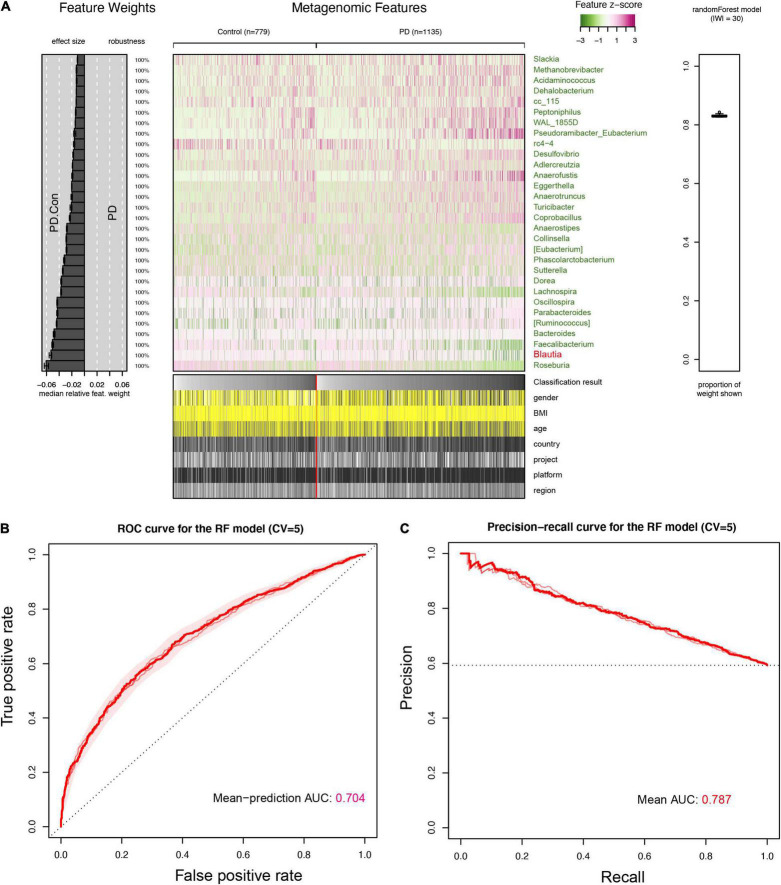
Interpretation and evaluation plot of the RF model in feces for detecting PD. An overview of the top 30 important features (genera) contributing to the predictive power of the random forest (RF) model for detecting PD were presented **(A)**. It showed that *Blautia* genus was the 2nd important feature for the RF model in discriminating PD patients from controls, and was also decreased in the feces of PD patients, which was same to results found in the brain and blood samples (Supplementary Figures 12, 13). The receiver operating characteristic curve (ROC) was plotted and AUC value from five folds cross-validation (CV = 5) was 0.704 **(B)**. The precision-recall Curve (PRC) and AUC value (0.787) was presented in **(C)**. The cases and controls in the RF were not evenly distributed, and the AUC value in PRC was higher than that in ROC.

To further manifest the role of *Blautia* genus in discriminating PD from control, we constructed a stratified 5-fold cross-validation RF model using the microbiota data of feces, blood, and brain, individually. The top 30 important genera contributing to the classifier (RF models, over 80% weights) in the feces were all decrease in PD. The *Blautia* genus was one of the important genera in this classifier (Figure 7A), which was further confirmed in the blood and brain (Supplementary Figures 12A, 13A). The classifier achieved an AUC of 0.704 and 0.787 for ROC and PRC in discriminating PD patients from controls (Figures 7B,C), respectively. The AUC value of ROC and PRC was 0.777 and 0.768 for the RF model using microbiota data from blood samples (Supplementary Figures 12B,C), and the AUC value of ROC and PRC was 0.689 and 0.701 for the RF model using microbiota data from brain samples (Supplementary Figures 13B,C).

## Discussion

Growing attention have been paid to evaluate the role of gut microbiota in PD, however, various alteration of microbiota composition has been reported without consistent conclusion (Hill-Burns et al., 2017; Heintz-Buschart et al., 2018; Lin et al., 2018). Recent studies have suggested that several bacteria could also be amplified in the blood and brain of PD patients and healthy controls (Qian et al., 2018a; Klann et al., 2020; Pisa et al., 2020), but little is known about the microbiota profile in PD patients’ blood and brain. In this study, we integrated the microbiota composition data from feces, blood, and brain samples using publicly available sequencing data. Our results first described the difference in microbiota composition among feces, blood, and brain samples. Furthermore, we found that *Blautia* was the unique genus commonly altered (depletion) among three sample types. In addition, *Blautia* genus was correlated with host brain genes involved in energy metabolism, protein degradation, and mitochondrial function, which were tightly implicated in the pathophysiology of PD. Finally, machine learning model (RF) on all three sample types also consistently showed that *Blautia* genus was one of the vital genera for the classifier to discriminate PD patients from controls.

In order to reduce the impact of environmental bacterial contamination, we removed all genera previously reported as potential contaminant or widely distributed in the tubes and agents before data analysis (Supplementary Data 1) (Salter et al., 2014; Davis et al., 2018; Eisenhofer et al., 2019). Our data showed that the ratio of F/B was increased in the feces of PD patients. It is reported that greater F/B ratio was associated with several diseases, including type 1 or type 2 diabetes mellitus (Larsen et al., 2010; Murri et al., 2013), obesity, and motor neuron disease (Turnbaugh et al., 2006; Ismail et al., 2011; Ngo et al., 2020). Indeed, a previous study showed that the F/B ratio in human fecal microbiota decreased with aging, which increased in adults while decreased in elderly individuals (Mariat et al., 2009), indicating that lower F/B ratio during aging might be a compensatory response in PD.

The overall results showed that the gut microbial α-diversity was increased in patients with PD at the genus level, which was consistent to the result reported in a latest published meta-analysis (Romano et al., 2021). It is generally considered that higher gut microbiota α-diversity is related to better gut function (Menni et al., 2020; Ortiz-Alvarez et al., 2020). The potential reason for the increased α-diversity in PD might derive from a decreased abundance of dominant species and an increased ratio of in rare/low abundant ones (Romano et al., 2021). The microbial β-diversity in the feces, but not in the blood and brain, was different between PD patients and controls, suggesting an obvious alteration of gut microbiota composition in PD.

Our results revealed that *Blautia* was the unique genus that significantly decreased across the feces, blood, and brain samples of PD patients. Due to the very low levels of microbiota in the blood and brain, it is worthy to note that only 14% blood samples and 68% brain samples had positive reads for *Blautia* genus, especially negative in patients with PD. To verify the stability of our results, we also applied zero-inflated regression model to re-calculate the data using pscl (V.1.5.5) package (Zeileis et al., 2008), and the results showed that PD patients were prone to having a lower abundance of *Blautia* genus than controls in blood (Count Estimate = −0.474, *P* = 0.0008; Zero-inflation Estimate = 1.801, *P* = 0.026) and brain (Count Estimate = −1.016, *P* < 2e-16; Zero-inflation Estimate = 0.497, *P* = 0.065). These data suggested that our results on *Blautia* genus distribution in PD patients and controls had a relatively good stability.

*Blautia* genus, belonging to the *Firmicutes* phylum, is a common gut microbial genus producing butyric acid and acetic acid (Liu X. et al., 2021). Previous studies have shown that the level of butyric acid and acetic acid was decreased in PD patients’ feces and blood (Unger et al., 2016; Aho et al., 2021; Wu et al., 2022). Recent studies showed that *Blautia* genus products could inhibit insulin signaling and fat accumulation in adipocytes by regulating G-protein coupled receptors (Benítez-Páez et al., 2020). It is reported that depletion of gut *Blautia* genus is tightly associated with the intestinal inflammation and worsening metabolic phenotype in children with obesity (Kimura et al., 2013; Benítez-Páez et al., 2020). In addition, *Blautia* genus could also alleviate the inflammatory diseases of eyes and exert probiotic activity against specific microorganisms. In the light of the above evidence, *Blautia* genus is considered to be a potential “probiotic bacterium” and play a beneficial role in human health. Thus, deletion of *Blautia* genus among gut, blood, and brain may be implicated in the pathogenesis of PD *via* regulating neuroinflammation and metabolism. A recent shotgun metagenome analysis of gut microbiome also showed that *Blautia* genus was significantly decreased in PD (Qian et al., 2020). At species level, our analysis using the above shotgun metagenome data showed that *Blautia argi, Blautia coccoides, Blautia sp. SC05B48, and Blautia hansenii* species were decreased in patients with PD (Supplementary Table 5). Further studies in animals and humans are required to deeply validate whether *Blautia* genus and species plays a role in the development and progression of PD.

In order to preliminarily explore the relationship between *Blautia* genus and PD, we performed a correlation analysis between host brain genes and *Blautia* genus identified from the same RNA-Seq data. There were nine genes (MALAT1, MAP1B, TULP4, ZNF221, TAOK1, RPS5, BCLAF3, GOLGB1, and TAF8) correlated to *Blautia* genus with an absolute *r* value greater than 0.45. Metastasis-associated lung adenocarcinoma transcript 1 (MALAT1) is the only lncRNA negatively correlated with *Blautia* genus abundance (*r* = −0.479). Recent studies demonstrated that MALAT1 could induce cell death and inflammation through regulating miR-124 function and suppressing nuclear factor erythroid 2-related factor 2 (NRF2) in PD (Liu et al., 2017; Cai et al., 2020; Lu et al., 2020), respectively. Microtubule associated protein 1B (MAP1B) is also negatively correlated with *Blautia* genus (*r* = −0.453). MAP1B is a critical part of the cytoskeleton, which plays a vital role in maintaining neuronal function (Teng et al., 2001). MAP1B has been reported to alleviate leucine-rich repeat kinase 2 (LRRK2) mutant-mediated neuronal damage in PD (Chan et al., 2014). Indeed, MAP1B dysfunction is also widely reported to be involved in the pathogenesis of AD (Gevorkian et al., 2008; Mitsuyama et al., 2018), which shared many common pathological changes with PD. However, the role of other seven top genes associated with *Blautia* genus in PD remains unclear.

We further performed a functional enrichment analysis individually using three different *Blautia*-correlated host gene sets, including whole host genes (*n* = 3583), DEGs (*n* = 1393) from RNA-Seq data, and overlapping DEGs (*n* = 36) between RNA-Seq and microarray data. The results showed that host genes significantly correlated with *Blautia* genus abundance were mainly targeted to pathways involved in mitochondrial terms, energy metabolism, and inflammatory response, which have been reported to be associated with the pathogenesis of PD (Rocha et al., 2018). Mitochondrial impairment is a vital pathological feature in PD (Rocha et al., 2018), suggesting mitochondrial alterations might mediate the association between *Blautia* genus and PD. GSEA-based KEGG analysis showed that *Blautia*-correlated host genes were mainly mapped to PD and other neurodegenerative diseases (NDDs), including AD, HD, ALS, and prion disease, which was further confirmed by the functional prediction data in PICRUSt2. However, the distribution and role of *Blautia* genus in other NDDs is still largely unknown. Interestingly, we found *Blautia*-correlated host genes were also targeted to metabolic diseases, such as DCM and NAFLD. Indeed, previous studies have demonstrated that the gut *Blautia* genus was strongly associated with obesity and diabetes milletus (Tong et al., 2018; Ozato et al., 2019), which was consistent to the results of our study. Although studies have pointed out a role of gut microbiota in NAFLD (Safari and Gérard, 2019; Aron-Wisnewsky et al., 2020), however, whether *Blautia* genus is involved in the energy metabolism impairment of NAFLD is still elusive.

Besides mitochondrial function, it is worth noting that the 36 *Blautia*-correlated DEGs (overlapping between RNA-Seq and microchip) were also enriched in pH regulation and protein degradation. Disorders of lysosomal acidification (pH increase) have been reported to impair the ability of lysosome in degradation of misfolded proteins (Bourdenx et al., 2016). Since abnormal alpha-synuclein aggregation induced by impaired proteasomal and lysosomal function is a vital pathological feature in PD (Chu et al., 2009; Hoffmann et al., 2019), further studies are warranted to test whether impairment of protein degradation pathway mediates the association between *Blautia* genus and alpha-synuclein clearance in PD. Moreover, as NDDs share several common pathogenetic mechanisms including misfolded proteins/peptides aggregation and chronic inflammation (Currais et al., 2017; Butnaru and Chapman, 2019), it inspires us to speculate that *Blautia* genus may probably exert a common effect in NDDs, which deserves to be investigated in future studies.

In summary, to the best of our knowledge, our study here first describes the microbiota landscape across the feces, blood, and brain samples of patients with PD and controls. Our key finding is that *Blautia* is the unique genus having the same downward trend in PD patients’ feces, blood, and brain. Moreover, *Blautia* genus is tightly correlated to host genes involved in energy metabolism, inflammatory response, and mitochondrial function, impairment of which are the critical pathophysiological features in PD. Our results reconfirm the microbiota dysbiosis in PD, and suggest that alterations of mitochondrial function and immune response may mediate the link between depletion of *Blautia* genus and PD pathogenesis. Further studies are greatly encouraged to evaluate the role of *Blautia* genus in PD and other neurodegenerative diseases.

### Limitations of the Study

Some limitations in our study should be addressed here. First, this is the first study using the Kraken2 to extract microbiota reads in the brain RNA-Seq data. Although Kraken2 has been applied to identify microbiota reads in tumor RNA-Seq data with high sensitivity and specificity (Poore et al., 2020), it is still hard to rule out the possibility of a false positive result owing to bacterial contamination. We have tried our best to remove the potential contamination genera to minimize the impact of bacterial contamination on the final results. Second, it is worthy to note there is obvious heterogeneity among the collected microbiome datasets across studies. As no optimal way is currently available to correct batch effects, only genus-level data was used in this study for microbiome analysis to alleviate heterogeneity (Wang et al., 2020). This leads to a loss of many OTUs, which may affect the results of diversity analysis. Third, microbiota identified in the blood and brain might originate from the bacterial debris, whose DNA/RNA can also transport in the blood and brain. However, there is no effective method now to distinguish them from live bacteria. Forth, the transcriptome and microbiota data obtained in this study were based on the same RNA-Seq data and the correlation threshold (*r* > 0.3) was relatively low, which may contribute to an over-estimation of the correlation and enrichment analysis. Fifth, since we use “pan-contaminants” to remove bacteria not found in the feces, these may result in the loss of a lot of information and introduce potential bias to the final results. Sixth, only one study was included for microbiota analysis in blood samples. Taken together, our results in this study need to be interpreted very cautiously.

## Data Availability Statement

The original contributions presented in the study are included in the article/Supplementary Material, further inquiries can be directed to the corresponding author.

## Author Contributions

XG, PT, YL, and RL conceived and designed the project. XG, CH, and LChe collected and analyzed the data. XG, XZ, and LCho drafted the manuscript. LZ, PL, and RL revised the manuscript. All authors approved the final manuscript.

## Conflict of Interest

The authors declare that the research was conducted in the absence of any commercial or financial relationships that could be construed as a potential conflict of interest.

## Publisher’s Note

All claims expressed in this article are solely those of the authors and do not necessarily represent those of their affiliated organizations, or those of the publisher, the editors and the reviewers. Any product that may be evaluated in this article, or claim that may be made by its manufacturer, is not guaranteed or endorsed by the publisher.

## Footnotes

1
https://www.ncbi.nlm.nih.gov/sra

2
https://www.ebi.ac.uk/ena/browser/home/

3
http://www.ilincs.org/apps/grein/?gse=

4
https://imageo.genyo.es/
