# Supplementary material for: Integrated Microbiome and Host Transcriptome Profiles Link Parkinson’s Disease to Blautia Genus: Evidence From Feces, Blood, and Brain
Source: Front Microbiol. 2022 May 26;13:875101. doi: 10.3389/fmicb.2022.875101 (PMC9204254; doi:10.3389/fmicb.2022.875101)
Supplement: Supplementary file 2 [file Table_1.DOCX]

**Supplementary Table 1. Summary of the brain microarray Projects included in the meta-analysis.**

| **Project** | **GSE ID** | **Platform** | **Tissue** | **Country** | **City** | **Con (n)** | **PD (n)** | **Article(s) PMID** |
| --- | --- | --- | --- | --- | --- | --- | --- | --- |
| PRJNA140647 | GSE28894 | GPL6104 | Brain | USA | Bethesda | 59 | 55 | NA * |
| PRJNA151407 | GSE34516 | GPL5175 | Brain | Spain | Barcelona | 4 | 4 | 22634372 |
| PRJNA212736 | GSE49036 | GPL570 | Brain | Netherlands | Amsterdam | 8 | 20 | 26087293 |
| PRJNA136075 | GSE26927 | GPL6255 | Brain | UK | London | 8 | 12 | 22864814, 25119539 |
| PRJNA122363 | GSE19587 | GPL571 | Brain | USA | New York | 10 | 12 | 20837543 |
| PRJNA125325 | GSE20295 | GPL96 | Brain | USA | Syracuse | 53 | 40 | 15965975, 20926834 |
| PRJNA125567 | GSE20146 | GPL570 | Brain | USA | Syracuse | 10 | 10 | 20926834 |
| PRJNA101445 | GSE8397 | GPL97 | Brain | UK | London | 18 | 29 | 16344956, 17211632 |
| PRJNA100241 | GSE7621 | GPL570 | Brain | USA | Gaithersburg | 9 | 16 | 17571925 |

* NA: No related article has been published until now.
