# Supplementary material for: Integrated Microbiome and Host Transcriptome Profiles Link Parkinson’s Disease to Blautia Genus: Evidence From Feces, Blood, and Brain
Source: Front Microbiol. 2022 May 26;13:875101. doi: 10.3389/fmicb.2022.875101 (PMC9204254; doi:10.3389/fmicb.2022.875101)
Supplement: Supplementary file 3 [file Table_2.DOCX]

**Supplementary Table 2. Summary of the KEGG pathway of Level 2 significantly altered between PD patients and controls.**

| **KEGG pathway Level 2** | **TE.fixed** | **lower.fixed** | **upper.fixed** | **pval.fixed** |
| --- | --- | --- | --- | --- |
| Excretory System | 0.085505896 | 0.031783839 | 0.139228 | 0.001811294 |
| Neurodegenerative Diseases | 0.034575522 | 0.000527645 | 0.0686234 | 0.04655374 |
| Transport and Catabolism | 0.027346061 | 0.010921733 | 0.04377039 | 0.00110128 |
| Cancers | 0.021353099 | 0.003166363 | 0.03953984 | 0.02138037 |
| Infectious Diseases | 0.016113703 | 0.00277414 | 0.02945327 | 0.01790559 |
| Signaling Molecules and Interaction | 0.015935088 | 0.002841273 | 0.0290289 | 0.0170669 |
| Nucleotide Metabolism | 0.014262649 | 0.007088167 | 0.02143713 | 9.7654E-05 |
| Amino Acid Metabolism | 0.012154902 | 0.00676425 | 0.01754555 | 9.89989E-06 |
| Genetic Information Processing | 0.011676056 | 0.003345828 | 0.02000629 | 0.006010987 |
| Metabolism of Terpenoids and Polyketides | 0.010958484 | 0.004544613 | 0.01737235 | 0.000811869 |
| Replication and Repair | 0.010947158 | 0.00390403 | 0.01799029 | 0.002316158 |
| Folding, Sorting and Degradation | -0.00559623 | -0.010392143 | -0.000800316 | 0.02219394 |
| Metabolism of Other Amino Acids | -0.007414169 | -0.014762258 | -6.60793E-05 | 0.04797505 |
| Metabolism of Cofactors and Vitamins | -0.010212582 | -0.017132408 | -0.003292757 | 0.003820665 |
| Glycan Biosynthesis and Metabolism | -0.023714143 | -0.044192821 | -0.003235466 | 0.0232305 |
| Biosynthesis of Other Secondary Metabolites | -0.030591872 | -0.044731266 | -0.01645248 | 2.22962E-05 |
| Cell Motility | -0.040235698 | -0.078049535 | -0.00242186 | 0.03702451 |
| Immune System Diseases | -0.07724072 | -0.106733545 | -0.0477479 | 2.85039E-07 |
