# Supplementary material for: Integrated Microbiome and Host Transcriptome Profiles Link Parkinson’s Disease to Blautia Genus: Evidence From Feces, Blood, and Brain
Source: Front Microbiol. 2022 May 26;13:875101. doi: 10.3389/fmicb.2022.875101 (PMC9204254; doi:10.3389/fmicb.2022.875101)
Supplement: Supplementary file 4 [file Table_3.DOCX]

**Supplementary Table 3. Summary of the KEGG pathway of Level 3 significantly altered between PD patients and controls.**

| **KEGG pathway Level 3** | **TE.fixed** | **lower.fixed** | **upper.fixed** | **pval.fixed** |
| --- | --- | --- | --- | --- |
| Alzheimer's disease | 0.027209915 | 0.008938903 | 0.045480928 | 0.003513185 |
| Aminoacyl-tRNA biosynthesis | 0.019264989 | 0.00644908 | 0.032080899 | 0.003216676 |
| Amoebiasis | 0.205469345 | 0.107714341 | 0.303224348 | 3.79514E-05 |
| Antigen processing and presentation | -0.016233415 | -0.032250717 | -0.000216113 | 0.04698796 |
| Apoptosis | -0.113407507 | -0.215774867 | -0.011040148 | 0.02990527 |
| Arginine and proline metabolism | -0.008522531 | -0.016098563 | -0.000946499 | 0.02746611 |
| Bacterial chemotaxis | -0.044571353 | -0.088215108 | -0.000927598 | 0.04532553 |
| Bacterial secretion system | 0.016959606 | 0.00388983 | 0.030029382 | 0.01098144 |
| Bacterial toxins | 0.023247635 | 0.004341572 | 0.042153698 | 0.0159504 |
| Basal transcription factors | 0.119172914 | 0.00955391 | 0.228791919 | 0.03310679 |
| Base excision repair | 0.02529836 | 0.01576438 | 0.03483234 | 1.98483E-07 |
| beta-Alanine metabolism | -0.06291575 | -0.081904238 | -0.043927261 | 8.3547E-11 |
| beta-Lactam resistance | -0.057665288 | -0.112759477 | -0.002571098 | 0.04022506 |
| Betalain biosynthesis | 0.399758677 | 0.057661061 | 0.741856293 | 0.02200284 |
| Biosynthesis of ansamycins | -0.027340252 | -0.052603456 | -0.002077049 | 0.03391295 |
| Biosynthesis of siderophore group nonribosomal peptides | -0.055050458 | -0.107697296 | -0.00240362 | 0.04041841 |
| Biosynthesis of vancomycin group antibiotics | -0.023711044 | -0.046170715 | -0.001251372 | 0.0385305 |
| Bladder cancer | 0.017918366 | 0.001225624 | 0.034611107 | 0.03538982 |
| Caffeine metabolism | -0.044173515 | -0.066820682 | -0.021526348 | 0.000131876 |
| Calcium signaling pathway | -0.031561107 | -0.04498423 | -0.018137984 | 4.0585E-06 |
| Carbohydrate metabolism | -0.021442321 | -0.039406019 | -0.003478624 | 0.01930926 |
| Carbon fixation pathways in prokaryotes | -0.01708531 | -0.030819502 | -0.003351119 | 0.01476094 |
| Cardiac muscle contraction | 0.018103374 | 0.004034729 | 0.032172019 | 0.011667 |
| Cell cycle - Caulobacter | -0.031151172 | -0.04719768 | -0.015104663 | 0.000141868 |
| Cell division | -0.017260831 | -0.032365759 | -0.002155903 | 0.02510997 |
| Cellular antigens | -0.028904405 | -0.042991325 | -0.014817486 | 5.78104E-05 |
| Chaperones and folding catalysts | -0.029718271 | -0.048596999 | -0.010839544 | 0.002033395 |
| Chloroalkane and chloroalkene degradation | -0.016619802 | -0.025576517 | -0.007663087 | 0.000275994 |
| Colorectal cancer | -0.041826746 | -0.068584435 | -0.015069058 | 0.002185802 |
| Cyanoamino acid metabolism | 0.017242326 | 0.007803467 | 0.026681185 | 0.000343146 |
| Cysteine and methionine metabolism | 0.025235937 | 0.001155104 | 0.049316771 | 0.03997772 |
| DNA replication proteins | 0.013341883 | 0.001547856 | 0.02513591 | 0.02661005 |
| Epithelial cell signaling in Helicobacter pylori infection | 0.036666219 | 0.008467958 | 0.06486448 | 0.01081741 |
| Ether lipid metabolism | -0.019982357 | -0.036480866 | -0.003483847 | 0.01760453 |
| Ethylbenzene degradation | -0.035096323 | -0.057003187 | -0.01318946 | 0.001689484 |
| Flavonoid biosynthesis | -0.049275381 | -0.074047261 | -0.024503502 | 9.6713E-05 |
| Fructose and mannose metabolism | -0.009843614 | -0.018123365 | -0.001563863 | 0.0197978 |
| Glioma | -0.018967437 | -0.027434761 | -0.010500114 | 1.13108E-05 |
| Glutathione metabolism | 0.016480709 | 0.005398811 | 0.027562606 | 0.003559083 |
| Glycolysis / Gluconeogenesis | -0.024684653 | -0.041033675 | -0.00833563 | 0.003083775 |
| Glycosaminoglycan degradation | -0.031527602 | -0.0466774 | -0.016377805 | 4.52691E-05 |
| Hepatitis C | 0.142452318 | 0.056897202 | 0.228007435 | 0.001100802 |
| Histidine metabolism | -0.034244552 | -0.062580948 | -0.005908157 | 0.0178547 |
| Homologous recombination | -0.020197314 | -0.037144308 | -0.003250321 | 0.01949791 |
| Isoflavonoid biosynthesis | 0.034535768 | 0.014947832 | 0.054123705 | 0.000548988 |
| Isoquinoline alkaloid biosynthesis | -0.055200735 | -0.101909434 | -0.008492037 | 0.02054178 |
| Linoleic acid metabolism | 0.02303217 | 0.002515657 | 0.043548683 | 0.02778651 |
| Lipid biosynthesis proteins | -0.020849286 | -0.036381213 | -0.005317359 | 0.008514451 |
| Long-term potentiation | -0.021224402 | -0.036278897 | -0.006169907 | 0.005723207 |
| Lysine degradation | 0.038746061 | 0.006494415 | 0.070997707 | 0.01854086 |
| N-Glycan biosynthesis | 0.012600287 | 0.000238263 | 0.024962311 | 0.04574486 |
| Nicotinate and nicotinamide metabolism | -0.019620904 | -0.033138316 | -0.006103493 | 0.004441936 |
| Novobiocin biosynthesis | -0.01854322 | -0.031426437 | -0.005660003 | 0.004786856 |
| Others | -0.020902989 | -0.039230893 | -0.002575086 | 0.025395 |
| Oxidative phosphorylation | -0.052062817 | -0.078813005 | -0.025312629 | 0.000136404 |
| p53 signaling pathway | -0.049656412 | -0.073769557 | -0.025543267 | 5.43306E-05 |
| Pancreatic secretion | -0.028332079 | -0.043444138 | -0.013220021 | 0.000238279 |
| Penicillin and cephalosporin biosynthesis | -0.028024692 | -0.051493119 | -0.004556266 | 0.01925896 |
| Pertussis | -0.023396845 | -0.044170774 | -0.002622917 | 0.02728409 |
| Phenylalanine metabolism | -0.015374159 | -0.029418818 | -0.0013295 | 0.03191299 |
| Phenylalanine, tyrosine and tryptophan biosynthesis | 0.018545627 | 0.005010207 | 0.032081046 | 0.007243112 |
| Prenyltransferases | 0.010505029 | 0.001948088 | 0.019061971 | 0.01612064 |
| Primary bile acid biosynthesis | 0.016389036 | 0.006403711 | 0.026374361 | 0.00129578 |
| Propanoate metabolism | 0.024765267 | 0.008791799 | 0.040738735 | 0.00237579 |
| Prostate cancer | -0.050207653 | -0.083341139 | -0.017074167 | 0.002978367 |
| Proteasome | -0.053424072 | -0.082417472 | -0.024430672 | 0.000304448 |
| Protein folding and associated processing | 0.029155851 | 0.010631889 | 0.047679813 | 0.002036263 |
| Purine metabolism | 0.022463604 | 0.0051073 | 0.039819908 | 0.01119006 |
| RNA degradation | -0.015917572 | -0.031649054 | -0.00018609 | 0.04735082 |
| RNA transport | -0.018782985 | -0.034698964 | -0.002867007 | 0.02072155 |
| Secondary bile acid biosynthesis | -0.017751954 | -0.032583934 | -0.002919974 | 0.01898515 |
| Steroid biosynthesis | 0.02664464 | 0.003874343 | 0.049414938 | 0.02182212 |
| Styrene degradation | -0.022064512 | -0.04034637 | -0.003782654 | 0.01800597 |
| Sulfur metabolism | -0.019437938 | -0.036350469 | -0.002525408 | 0.02428254 |
| Terpenoid backbone biosynthesis | -0.018694865 | -0.032661192 | -0.004728538 | 0.008702042 |
| Tetracycline biosynthesis | -0.022644845 | -0.042660323 | -0.002629368 | 0.02659317 |
| Transcription factors | 0.028293575 | 0.009614645 | 0.046972504 | 0.002989452 |
| Translation proteins | 0.039442392 | 0.007164929 | 0.071719855 | 0.01661876 |
| Transporters | -0.035142056 | -0.056717309 | -0.013566804 | 0.001410884 |
| Type I diabetes mellitus | -0.040544733 | -0.059630354 | -0.021459111 | 3.13174E-05 |
| Ubiquitin system | -0.081989789 | -0.13442336 | -0.029556218 | 0.002178347 |
| Vibrio cholerae infection | 0.093994464 | 0.019097251 | 0.168891677 | 0.01390476 |
| Vitamin B6 metabolism | -0.0329653 | -0.065817384 | -0.000113216 | 0.04921568 |
