# Supplementary material for: Integrated Microbiome and Host Transcriptome Profiles Link Parkinson’s Disease to Blautia Genus: Evidence From Feces, Blood, and Brain
Source: Front Microbiol. 2022 May 26;13:875101. doi: 10.3389/fmicb.2022.875101 (PMC9204254; doi:10.3389/fmicb.2022.875101)
Supplement: Supplementary file 5 [file Table_4.DOCX]

**Supplementary Table 4. Summary of the significantly changed fecal genera classified by Greengenes in the meta-analysis.**

| **Genera** | **TE.fixed** | **lower.fixed** | **upper.fixed** | **pval.fixed** |
| --- | --- | --- | --- | --- |
| Halomonas | 1.808420788 | 0.240541526 | 3.376300049 | 0.023780411 |
| Varibaculum | 1.218916925 | 0.507135387 | 1.930698462 | 0.000789604 |
| Citrobacter | 2.26867224 | 0.159347175 | 4.377997305 | 0.035028585 |
| Sutterella | -0.285705619 | -0.503034377 | -0.068376861 | 0.009977277 |
| Campylobacter | 0.795187737 | 0.213880063 | 1.376495411 | 0.007338235 |
| Methanobrevibacter | 1.187890323 | 0.665407297 | 1.710373348 | 8.35E-06 |
| Methanomassiliicoccus | 1.820387933 | 0.018924929 | 3.621850937 | 0.04764131 |
| Acidaminococcus | 0.907527104 | 0.203931204 | 1.611123003 | 0.011470042 |
| Phascolarctobacterium | -0.543394446 | -0.823408405 | -0.263380486 | 0.000142665 |
| Synergistes | 1.882167704 | 1.085261447 | 2.679073961 | 3.67E-06 |
| Dehalobacterium | 0.528162259 | 0.089919913 | 0.966404605 | 0.018170984 |
| rc4-4 | -0.84813561 | -1.389656673 | -0.306614548 | 0.002142632 |
| Anaerofustis | 1.378913765 | 0.854097976 | 1.903729555 | 2.61E-07 |
| Pseudoramibacter_Eubacterium | 1.640298176 | 1.035362074 | 2.245234277 | 1.07E-07 |
| [Eubacterium] | 0.273503826 | 0.006732242 | 0.54027541 | 0.044492018 |
| cc_115 | 0.837660288 | 0.317137637 | 1.358182938 | 0.001609902 |
| Turicibacter | 0.626330431 | 0.202118547 | 1.050542315 | 0.003806084 |
| Coprobacillus | 1.173780614 | 0.796419471 | 1.551141756 | 1.08E-09 |
| WAL_1855D | 1.665925298 | 1.128063328 | 2.203787268 | 1.27E-09 |
| 1-68 | 1.335962163 | 0.763382838 | 1.908541487 | 4.81E-06 |
| Finegoldia | 0.859617203 | 0.304047373 | 1.415187032 | 0.002424601 |
| ph2 | 0.767504463 | 0.066090028 | 1.468918898 | 0.031981681 |
| Peptoniphilus | 1.142977871 | 0.682045942 | 1.603909799 | 1.17E-06 |
| Anaerotruncus | 0.784671855 | 0.396582806 | 1.172760905 | 7.41E-05 |
| [Prevotella] | -1.600963741 | -2.862674109 | -0.339253374 | 0.012883757 |
| Bacteroides | -0.303534158 | -0.405154569 | -0.201913746 | 4.79E-09 |
| Lachnospira | -0.317012554 | -0.532755825 | -0.101269283 | 0.003977186 |
| Blautia | -0.33388844 | -0.454534622 | -0.213242259 | 5.82E-08 |
| Roseburia | -0.807595905 | -1.082617383 | -0.532574427 | 8.64E-09 |
| [Ruminococcus] | 0.244061053 | 0.080753957 | 0.407368149 | 0.003398911 |
| Dorea | 0.184136519 | 0.034155527 | 0.334117512 | 0.01611453 |
| Faecalibacterium | -0.431644111 | -0.609645204 | -0.253643017 | 2.01E-06 |
