# Supplementary material for: Integrated Microbiome and Host Transcriptome Profiles Link Parkinson’s Disease to Blautia Genus: Evidence From Feces, Blood, and Brain
Source: Front Microbiol. 2022 May 26;13:875101. doi: 10.3389/fmicb.2022.875101 (PMC9204254; doi:10.3389/fmicb.2022.875101)
Supplement: Supplementary file 6 [file Table_5.DOCX]

**Supplementary Table 5. Summary of the significantly changed fecal genera classified by SILVA in the meta-analysis.**

| **Silva_Genus** | **TE.fixed** | **lower.fixed** | **upper.fixed** | **pval.fixed** |
| --- | --- | --- | --- | --- |
| Methanobrevibacter | 1.1415532 | 0.621562067 | 1.661544321 | 1.68675E-05 |
| Phascolarctobacterium | -0.4727056 | -0.759063058 | -0.186348225 | 0.001214646 |
| Acidaminococcus | 0.9947841 | 0.269355752 | 1.720212504 | 0.007194386 |
| Campylobacter | 0.8166749 | 0.185063761 | 1.448285972 | 0.01126919 |
| Sutterella | -0.3591393 | -0.697411358 | -0.020867331 | 0.03744578 |
| Citrobacter | 1.1525409 | 0.272346523 | 2.032735311 | 0.01027576 |
| Proteus | 1.5711819 | 0.173196048 | 2.969167766 | 0.02761002 |
| Varibaculum | 1.4410153 | 0.672266821 | 2.209763718 | 0.000238836 |
| Bacteroides | -0.2700428 | -0.373024463 | -0.167061179 | 2.7547E-07 |
| Gallicola | 1.7374182 | 0.003543977 | 3.471292413 | 0.04953356 |
| Finegoldia | 0.9995147 | 0.410952893 | 1.588076417 | 0.000873231 |
| Peptoniphilus | 1.1217104 | 0.638713229 | 1.604707537 | 5.3186E-06 |
| Mogibacterium | 1.1406654 | 0.405337553 | 1.875993154 | 0.002362936 |
| Turicibacter | 0.515742 | 0.073395803 | 0.958088178 | 0.02230293 |
| Pediococcus | 2.0673942 | 0.272382419 | 3.862406042 | 0.02398453 |
| Anaerofustis | 1.290584 | 0.756331766 | 1.824836153 | 2.19432E-06 |
| Anaerotruncus | 0.6620992 | 0.336940971 | 0.987257336 | 6.58088E-05 |
| Faecalibacterium | -0.4694353 | -0.649930454 | -0.288940058 | 3.44149E-07 |
| Anaerostipes | -0.5434934 | -0.766306958 | -0.320679934 | 1.74596E-06 |
| Lachnospira | -0.650104 | -0.940387319 | -0.359820631 | 1.13645E-05 |
| Roseburia | -0.7222917 | -0.918175479 | -0.526407971 | 4.93523E-13 |
| Blautia | -0.3987374 | -0.521317503 | -0.276157381 | 1.82349E-10 |
| Dorea | -0.2334064 | -0.466498227 | -0.000314511 | 0.04969167 |
