# Supplementary material for: Integrated Microbiome and Host Transcriptome Profiles Link Parkinson’s Disease to Blautia Genus: Evidence From Feces, Blood, and Brain
Source: Front Microbiol. 2022 May 26;13:875101. doi: 10.3389/fmicb.2022.875101 (PMC9204254; doi:10.3389/fmicb.2022.875101)
Supplement: Supplementary file 7 [file Table_6.DOCX]

**Supplementary Table 6. Summary of the significantly changed genera in the blood.**

| **Genera** | **baseMean** | **log2FoldChange** | **lfcSE** | **Stat** | **Pvalue^*^** | **u95ci** | **l95ci** |
| --- | --- | --- | --- | --- | --- | --- | --- |
| Nesterenkonia | 71.80631967 | -2.483431825 | 0.759506146 | -3.269798193 | 0.001076242 | -0.994799778 | -3.972063872 |
| Meiothermus | 4.170637594 | -4.665919093 | 1.841216465 | -2.534150211 | 0.01127204 | -1.057134821 | -8.274703364 |
| Blautia | 2.468021626 | -3.811089996 | 1.660057449 | -2.295757896 | 0.021689728 | -0.557377397 | -7.064802596 |
| Dorea | 2.143825463 | -3.646055967 | 1.617308423 | -2.254397439 | 0.024171176 | -0.476131458 | -6.815980477 |

*: pvalue: not adjust pvalue
