# Supplementary material for: Integrated Microbiome and Host Transcriptome Profiles Link Parkinson’s Disease to Blautia Genus: Evidence From Feces, Blood, and Brain
Source: Front Microbiol. 2022 May 26;13:875101. doi: 10.3389/fmicb.2022.875101 (PMC9204254; doi:10.3389/fmicb.2022.875101)
Supplement: Supplementary file 8 [file Table_7.DOCX]

**Supplementary Table 7. Summary of the significantly changed genera in the brain.**

| **Genera** | **TE.fixed** | **lower.fixed** | **upper.fixed** | **pval.fixed** |
| --- | --- | --- | --- | --- |
| Bacteroides | -0.663059275 | -1.10356816 | -0.22255039 | 0.00317608 |
| Blautia | -0.772509225 | -1.338249635 | -0.206768815 | 0.007444029 |
| Thermoanaerobacterium | -1.409094888 | -2.003723768 | -0.814466007 | 3.41E-06 |
| Nocardioides | 0.630754553 | 0.318574845 | 0.942934262 | 7.49E-05 |
| Planctomyces | 0.806800608 | 0.067444872 | 1.546156344 | 0.032455586 |
| Rathayibacter | 0.387440029 | 0.09817923 | 0.676700828 | 0.008659716 |
| Syntrophobacter | 1.251943343 | 0.483725213 | 2.020161473 | 0.001402686 |
| Pseudonocardia | -0.658960074 | -1.190014023 | -0.127906124 | 0.015014552 |
| Elizabethkingia | -0.464947889 | -0.867322067 | -0.062573712 | 0.023527384 |
| Erythrobacter | -1.21729012 | -1.742610865 | -0.691969376 | 5.58E-06 |
| Halomonas | -0.394197901 | -0.747930647 | -0.040465156 | 0.028949501 |
| Cronobacter | 1.560703249 | 0.362918582 | 2.758487916 | 0.010654942 |
| Finegoldia | -0.733861308 | -1.348952284 | -0.118770331 | 0.019365413 |
