# Supplementary material for: Integrated Microbiome and Host Transcriptome Profiles Link Parkinson’s Disease to Blautia Genus: Evidence From Feces, Blood, and Brain
Source: Front Microbiol. 2022 May 26;13:875101. doi: 10.3389/fmicb.2022.875101 (PMC9204254; doi:10.3389/fmicb.2022.875101)
Supplement: Supplementary file 9 [file Table_8.DOCX]

**Supplementary Table 8. Summary of the significantly changed Blautia species using fecal shotgun metagenome data.**

| **Species** | **baseMean** | **log2FoldChange** | **LfcSE** | **Stat** | **Pvalue** | **U95ci** | **L95ci** |
| --- | --- | --- | --- | --- | --- | --- | --- |
| Blautia argi | 6014.396 | -1.2295347 | 0.2924155 | -4.204752 | 2.61369E-05 | -0.65640029 | -1.8026691 |
| Blautia coccoides | 1.93174 | -1.0415255 | 0.3890029 | -2.677424 | 0.007419078 | -0.27907983 | -1.8039711 |
| Blautia sp. SC05B48 | 18511.37 | -0.6438162 | 0.2415658 | -2.665179 | 0.007694728 | -0.17034722 | -1.1172853 |
| Blautia hansenii | 5862.087 | -0.6540903 | 0.2560668 | -2.554373 | 0.01063791 | -0.15219932 | -1.1559813 |
| Blautia sp. LZLJ-3 | 1557.817 | -0.4152598 | 0.2228714 | -1.863227 | 0.0624304 | 0.02156803 | -0.8520877 |
| Blautia obeum | 21188.93 | -0.3388337 | 0.1913349 | -1.770893 | 0.07657848 | 0.03618276 | -0.7138501 |
| Blautia sp. YL58 | 0.3994418 | -1.6163654 | 1.1746155 | -1.37608 | 0.1687967 | 0.68588106 | -3.9186119 |
| Blautia producta | 7938.43 | -0.3828278 | 0.2909619 | -1.315732 | 0.1882642 | 0.18745758 | -0.9531131 |

*: pvalue: not adjusted pvalue
