# Supplementary material for: Integrated Microbiome and Host Transcriptome Profiles Link Parkinson’s Disease to Blautia Genus: Evidence From Feces, Blood, and Brain
Source: Front Microbiol. 2022 May 26;13:875101. doi: 10.3389/fmicb.2022.875101 (PMC9204254; doi:10.3389/fmicb.2022.875101)
Supplement: Supplementary file 10 [file Table_9.DOCX]

**Supplementary Table 9. Summary of the gene/transcript biotypes of the DEGs (RNA-Seq) significantly associated with *Blautia* genus (|r| > 0.3 & p < 0.05).**

| Gene_biotype | DOWN | UP |
| --- | --- | --- |
| lncRNA | 20 | 58 |
| miRNA | 4 | 14 |
| misc_RNA | 1 | 2 |
| processed_pseudogene | 1 | 0 |
| protein_coding | 449 | 670 |
| scaRNA | 0 | 1 |
| snoRNA | 3 | 1 |
| snRNA | 1 | 1 |
| transcribed_processed_pseudogene | 1 | 2 |
| transcribed_unitary_pseudogene | 0 | 2 |
| transcribed_unprocessed_pseudogene | 5 | 6 |
| undefined | 84 | 67 |
