# Supplementary material for: Integrated Microbiome and Host Transcriptome Profiles Link Parkinson’s Disease to Blautia Genus: Evidence From Feces, Blood, and Brain
Source: Front Microbiol. 2022 May 26;13:875101. doi: 10.3389/fmicb.2022.875101 (PMC9204254; doi:10.3389/fmicb.2022.875101)
Supplement: Supplementary file 11 [file Image_1.PDF]

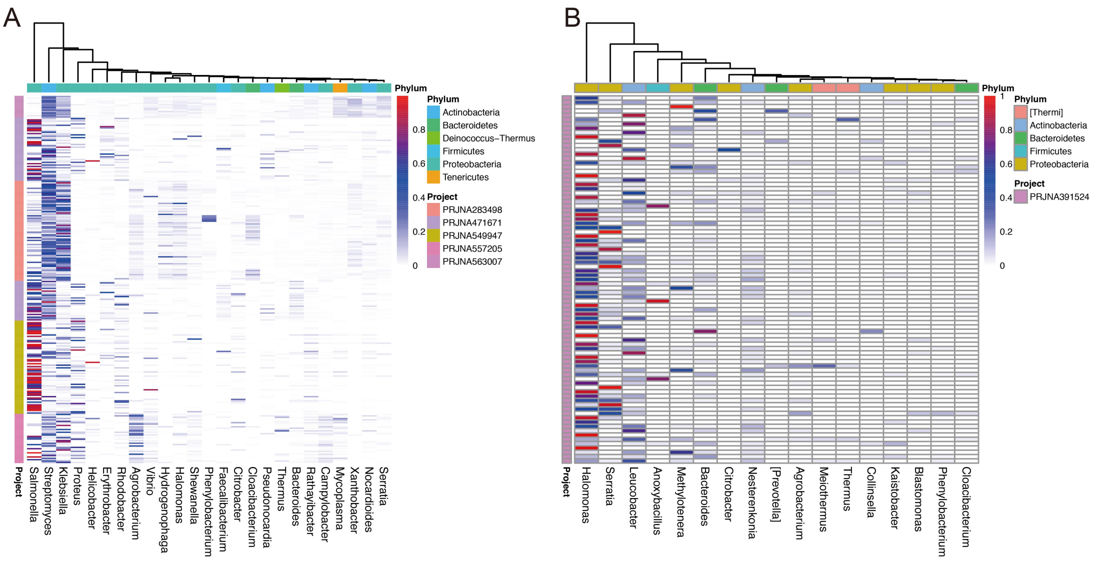

Supplementary Figure 1. Heatmap showing microbiota composition at the genus level in the brain and blood of PD patients. 25 genera in brain (**A**) and 17 genera in blood (**B**) with average relative abundance greater than 0.5% across all samples were shown. The genera presented here were filtered with previously reported potential contaminant bacteria. Those genera not appearing in the fecal microbiome were also removed (pan-contaminants).
