# Supplementary material for: Integrated Microbiome and Host Transcriptome Profiles Link Parkinson’s Disease to Blautia Genus: Evidence From Feces, Blood, and Brain
Source: Front Microbiol. 2022 May 26;13:875101. doi: 10.3389/fmicb.2022.875101 (PMC9204254; doi:10.3389/fmicb.2022.875101)
Supplement: Supplementary file 12 [file Image_2.PDF]

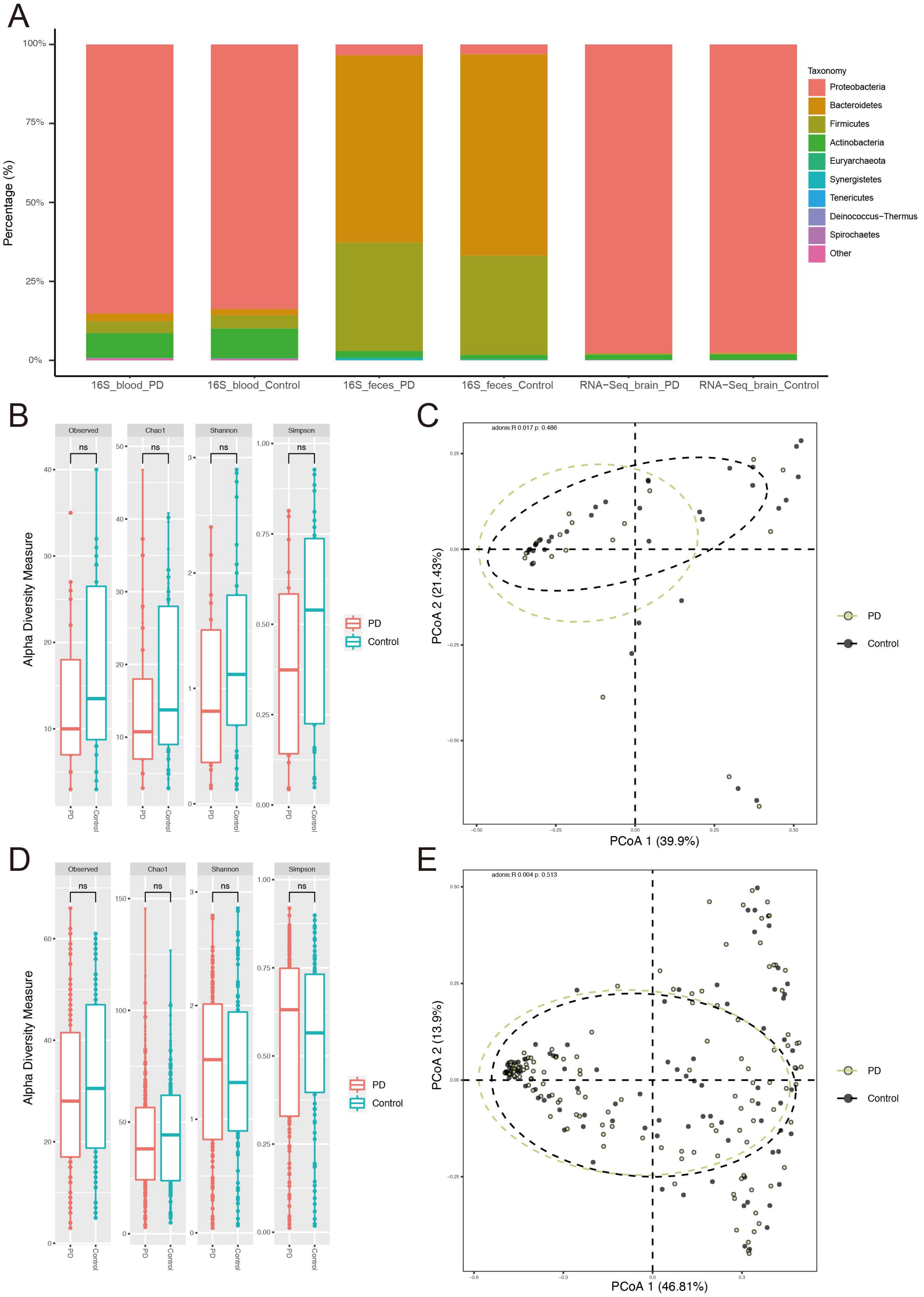

Supplementary Figure 2. Descriptions of microbial composition and diversity at the genus level in the blood and brain of PD patients and controls. A showed the microbiota composition among feces, blood and

brain samples in PD and control at genus level after removing pan-contaminants. Almost all the *Firmicutes* Phylum found in brain were not presented in fecal samples. There was no obvious difference in α-diversity (Observed, Shannon, Simpson and Chao1 indices) (**B**) and β-diversity (Bray distance) (**C**) of microbial communities in the blood between PD patients and controls. No significant difference was found of both α-diversity (Observed, Shannon, Simpson and Chao1) (**D**) and β-diversity (Bray distance) (**E**) in the brain.
