# Supplementary material for: Integrated Microbiome and Host Transcriptome Profiles Link Parkinson’s Disease to Blautia Genus: Evidence From Feces, Blood, and Brain
Source: Front Microbiol. 2022 May 26;13:875101. doi: 10.3389/fmicb.2022.875101 (PMC9204254; doi:10.3389/fmicb.2022.875101)
Supplement: Supplementary file 13 [file Image_3.PDF]

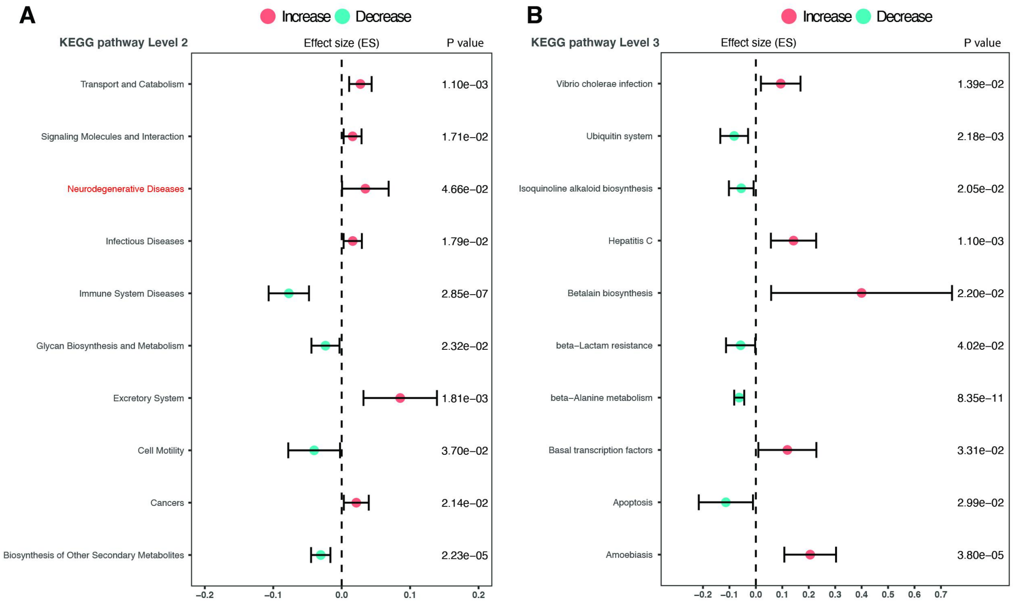

Supplementary Figure 3. KEGG pathway analysis of functional prediction data produced by PICRUSt2. The top ten altered KEGG pathways of Level 2 were presented in **A**. The top ten altered KEGG pathways of Level 3 were presented in **B**.
