# Supplementary material for: Integrated Microbiome and Host Transcriptome Profiles Link Parkinson’s Disease to Blautia Genus: Evidence From Feces, Blood, and Brain
Source: Front Microbiol. 2022 May 26;13:875101. doi: 10.3389/fmicb.2022.875101 (PMC9204254; doi:10.3389/fmicb.2022.875101)
Supplement: Supplementary file 14 [file Image_4.PDF]

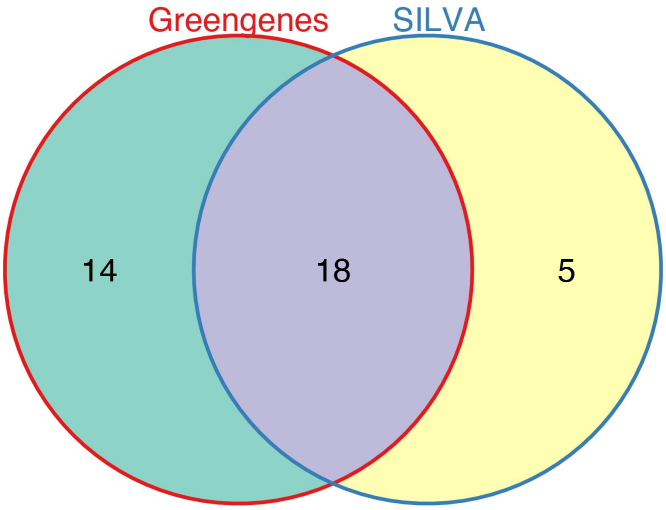

Supplementary Figure 4. Different gut microbiota genera found in microbiome analysis of 16S rRNA data using Greengenes and SILVA as reference, respectively.
