# Supplementary material for: Integrated Microbiome and Host Transcriptome Profiles Link Parkinson’s Disease to Blautia Genus: Evidence From Feces, Blood, and Brain
Source: Front Microbiol. 2022 May 26;13:875101. doi: 10.3389/fmicb.2022.875101 (PMC9204254; doi:10.3389/fmicb.2022.875101)
Supplement: Supplementary file 15 [file Image_5.PDF]

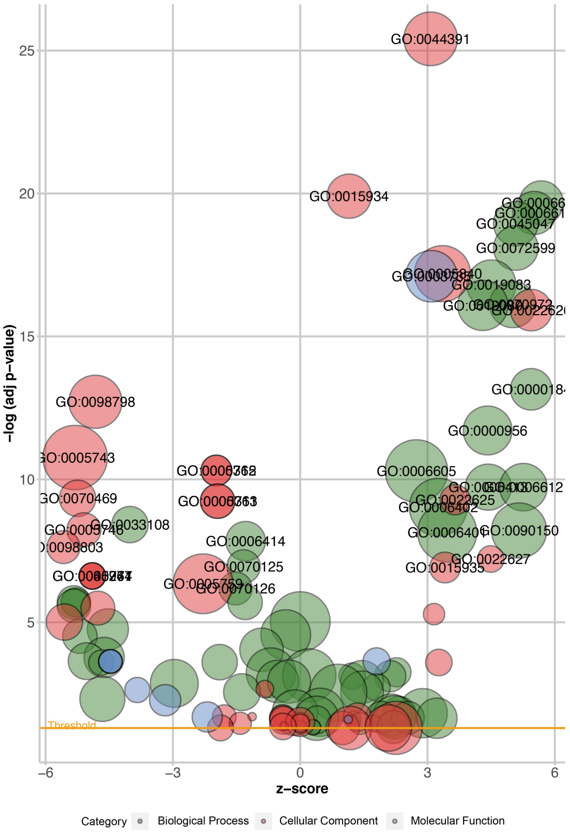

ID	Description
GO:0006613	cotranslational protein targeting to membrane
GO:0006614	SRP−dependent cotranslational protein targeting to membrane
GO:0045047	protein targeting to ER
GO:0072599	establishment of protein localization to endoplasmic reticulum
GO:0019083	viral transcription
GO:0070972	protein localization to endoplasmic reticulum
GO:0019080	viral gene expression
GO:0000184	nuclear−transcribed mRNA catabolic process, nonsense−mediated decay
GO:0000956	nuclear−transcribed mRNA catabolic process
GO:0006605	protein targeting
GO:0006413	translational initiation
GO:0006612	protein targeting to membrane
GO:0006402	mRNA catabolic process
GO:0033108	mitochondrial respiratory chain complex assembly
GO:0090150	establishment of protein localization to membrane
GO:0006401	RNA catabolic process
GO:0006414	translational elongation
GO:0070125	mitochondrial translational elongation
GO:0070126	mitochondrial translational termination
GO:0044391	ribosomal subunit
GO:0015934	large ribosomal subunit
GO:0005840	ribosome
GO:0022626	cytosolic ribosome
GO:0098798	mitochondrial protein complex
GO:0005743	mitochondrial inner membrane
GO:0000315	organellar large ribosomal subunit
GO:0005762	mitochondrial large ribosomal subunit
GO:0070469	respirasome
GO:0022625	cytosolic large ribosomal subunit
GO:0000313	organellar ribosome
GO:0005761	mitochondrial ribosome
GO:0005746	mitochondrial respirasome
GO:0098803	respiratory chain complex
GO:0022627	cytosolic small ribosomal subunit
GO:0015935	small ribosomal subunit
GO:0005747	mitochondrial respiratory chain complex I
GO:0030964	NADH dehydrogenase complex
GO:0045271	respiratory chain complex I
GO:0005759	mitochondrial matrix
GO:0003735	structural constituent of ribosome

Supplementary Figure 5. GO enrichment analysis of genes significantly associated with *Blautia* genus ($|r| > 0.3$ & $p < 0.05$) in PD brain. It showed that genes correlated to *Blautia* genus abundance were mainly mapped to mitochondrial function, proteins transportation and energy metabolism items.
