# Supplementary material for: Integrated Microbiome and Host Transcriptome Profiles Link Parkinson’s Disease to Blautia Genus: Evidence From Feces, Blood, and Brain
Source: Front Microbiol. 2022 May 26;13:875101. doi: 10.3389/fmicb.2022.875101 (PMC9204254; doi:10.3389/fmicb.2022.875101)
Supplement: Supplementary file 16 [file Image_6.PDF]

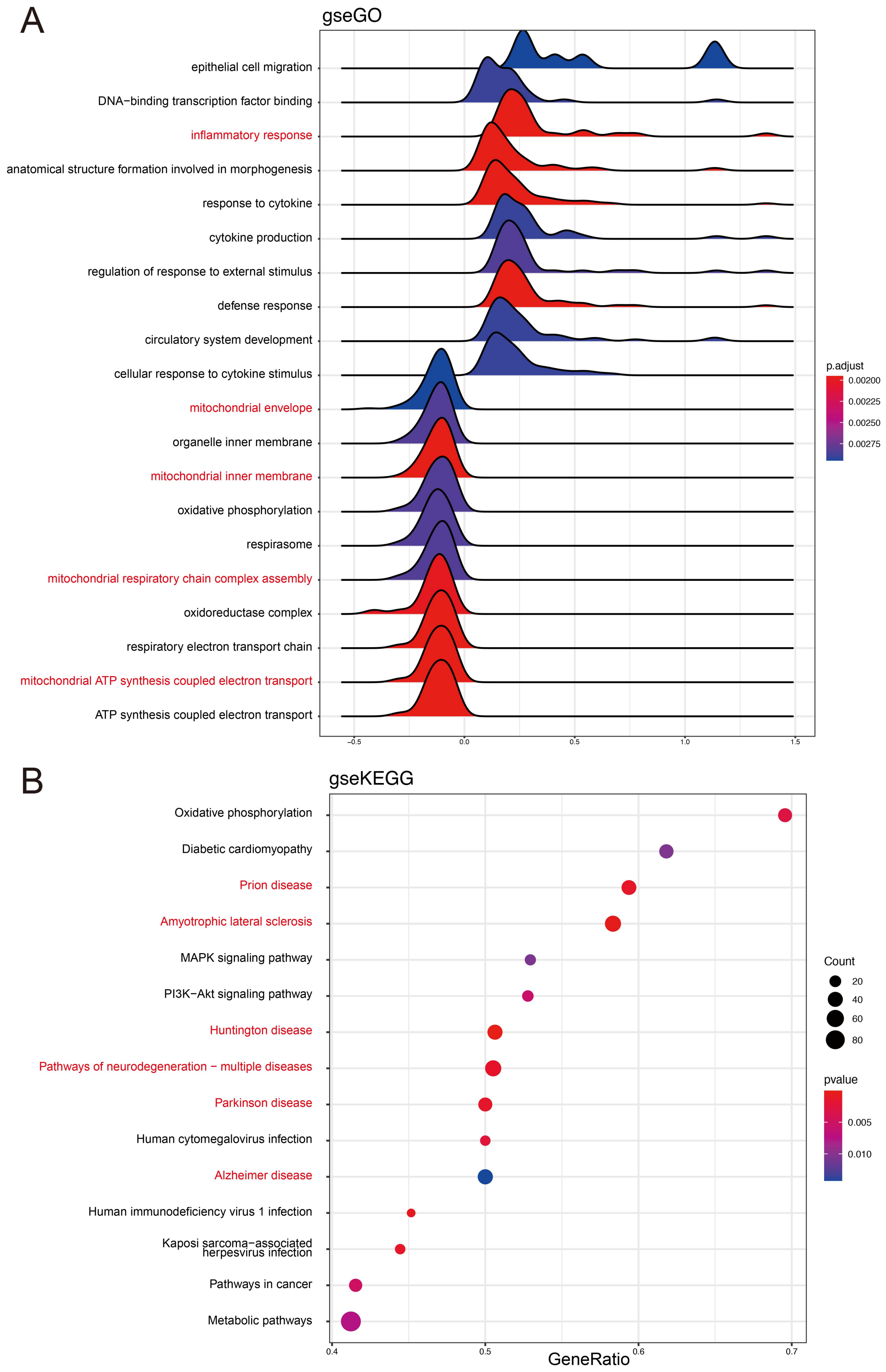

Supplementary Figure 6. GSEA based GO and KEGG enrichment

analysis of genes significantly associated with *Blautia* genus (|r| > 0.3 & p < 0.05) in PD brain. The gseGO result showed that *Blautia* genus correlated genes participating in inflammatory response were increased, but decreased in mitochondrial associated pathways (**A**). The gseKEGG result showed that *Blautia* genus related genes were mainly mapped to pathways involved in neurodegenerative diseases, including PD, (Amyotrophic Lateral Sclerosis) ALS, (Huntington's disease) HD and (Alzheimer's disease) AD, labeled in red (**B**).
