# Supplementary material for: Integrated Microbiome and Host Transcriptome Profiles Link Parkinson’s Disease to Blautia Genus: Evidence From Feces, Blood, and Brain
Source: Front Microbiol. 2022 May 26;13:875101. doi: 10.3389/fmicb.2022.875101 (PMC9204254; doi:10.3389/fmicb.2022.875101)
Supplement: Supplementary file 17 [file Image_7.PDF]

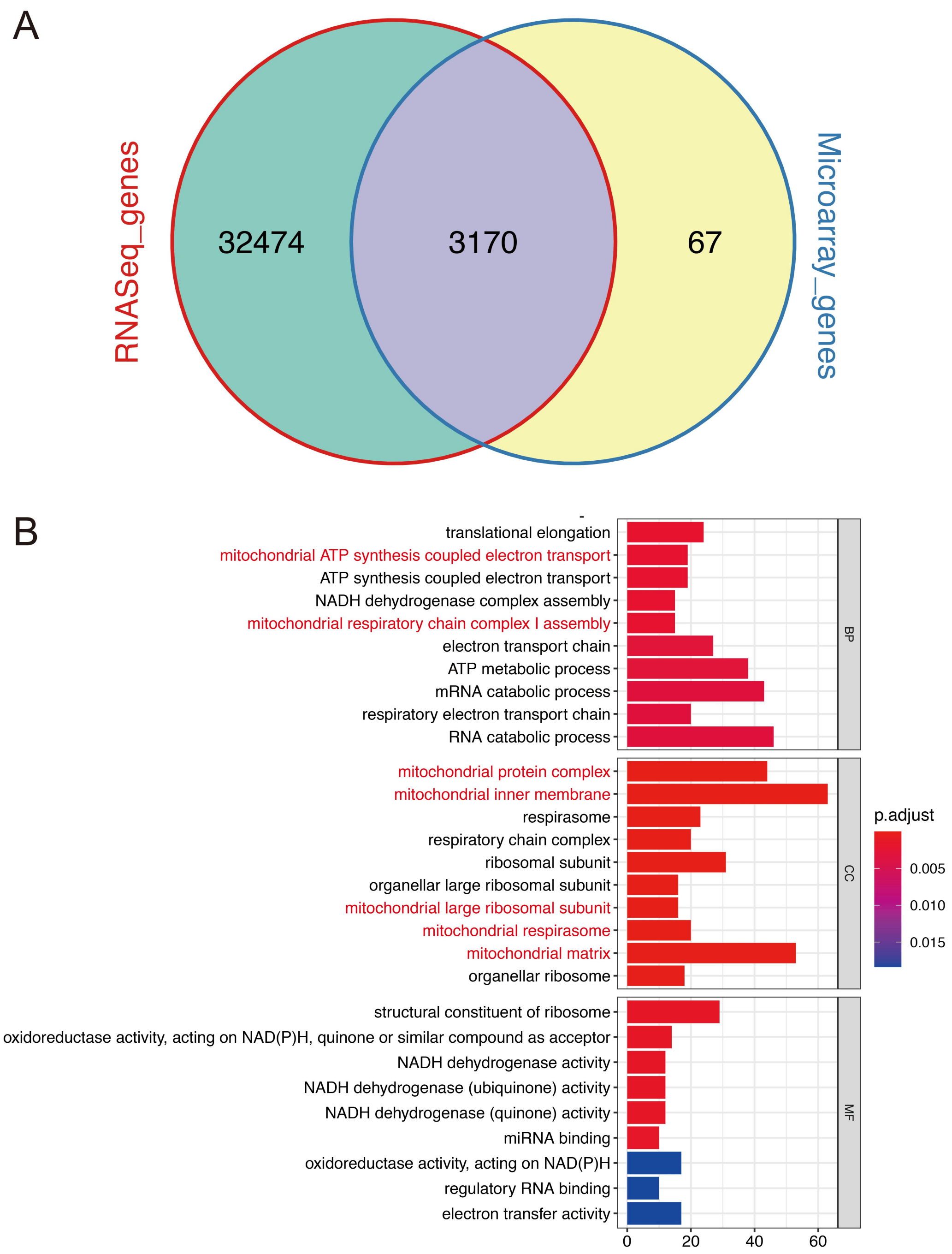

Supplementary Figure 7. GO enrichment analysis of DEGs significantly associated with *Blautia* genus. A showed the overlapped genes found in both RNA-Seq and microarray of brain samples, and over 98% genes identified in microarray data were covered in RNA-Seq data (**A**). **B** presented the results of GO enrichment analysis showing that

DEGs identified from RNA-Seq were significantly associated with *Blautia* genus ($|r| > 0.3$ & $p < 0.05$). These DEGs mainly participated in energy metabolism and mitochondrial function (red words).
