# Supplementary material for: Integrated Microbiome and Host Transcriptome Profiles Link Parkinson’s Disease to Blautia Genus: Evidence From Feces, Blood, and Brain
Source: Front Microbiol. 2022 May 26;13:875101. doi: 10.3389/fmicb.2022.875101 (PMC9204254; doi:10.3389/fmicb.2022.875101)
Supplement: Supplementary file 18 [file Image_8.PDF]

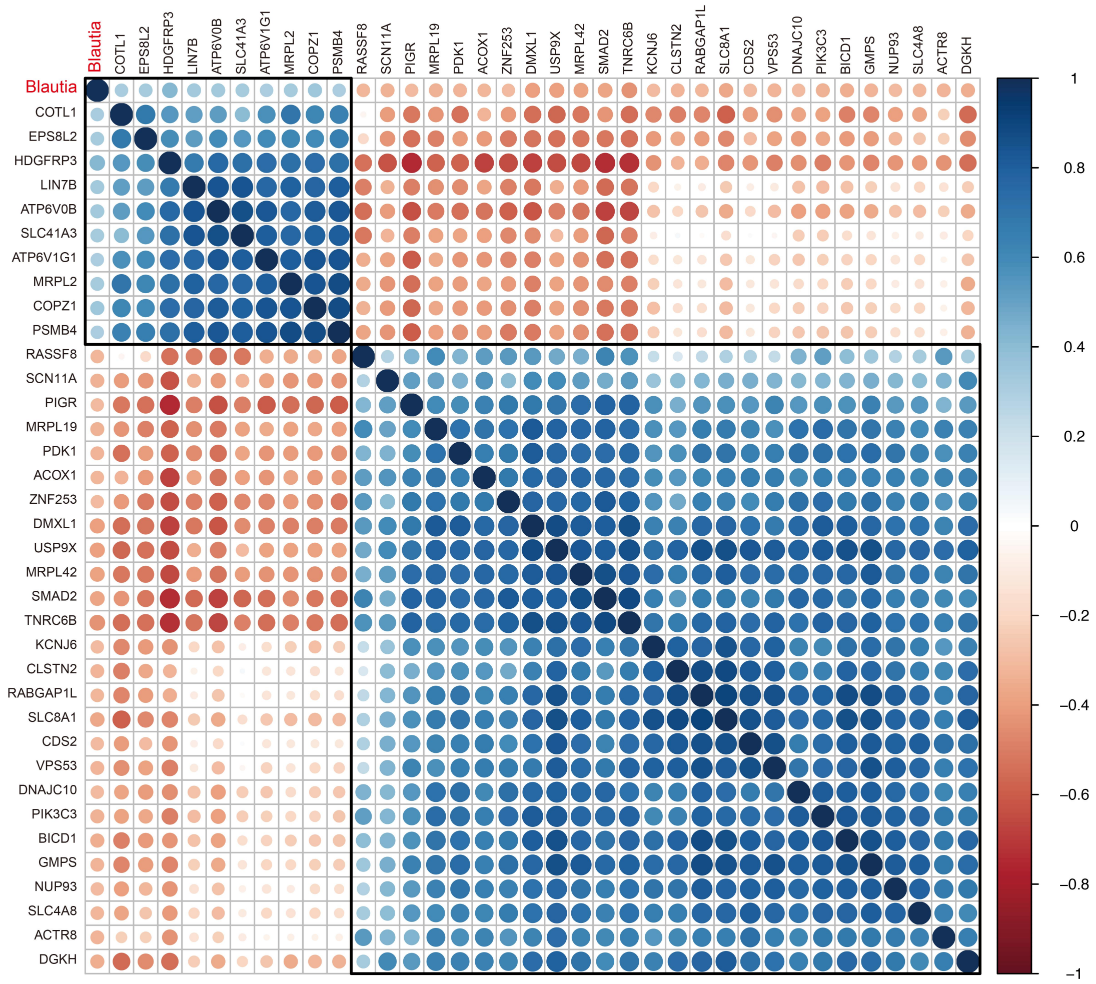

Supplementary Figure 8. Correlation matrix between *Blautia* genus and DEGs overlapped between RNA-Seq and microarray data. The correlation matrix (Spearman) showed that DEGs associated with *Blautia* genus ($|r| > 0.3$ & $p < 0.05$) mainly (about 2/3) had a negative relationship with the abundance of *Blautia* genus.
