# Supplementary material for: Integrated Microbiome and Host Transcriptome Profiles Link Parkinson’s Disease to Blautia Genus: Evidence From Feces, Blood, and Brain
Source: Front Microbiol. 2022 May 26;13:875101. doi: 10.3389/fmicb.2022.875101 (PMC9204254; doi:10.3389/fmicb.2022.875101)
Supplement: Supplementary file 19 [file Image_9.PDF]

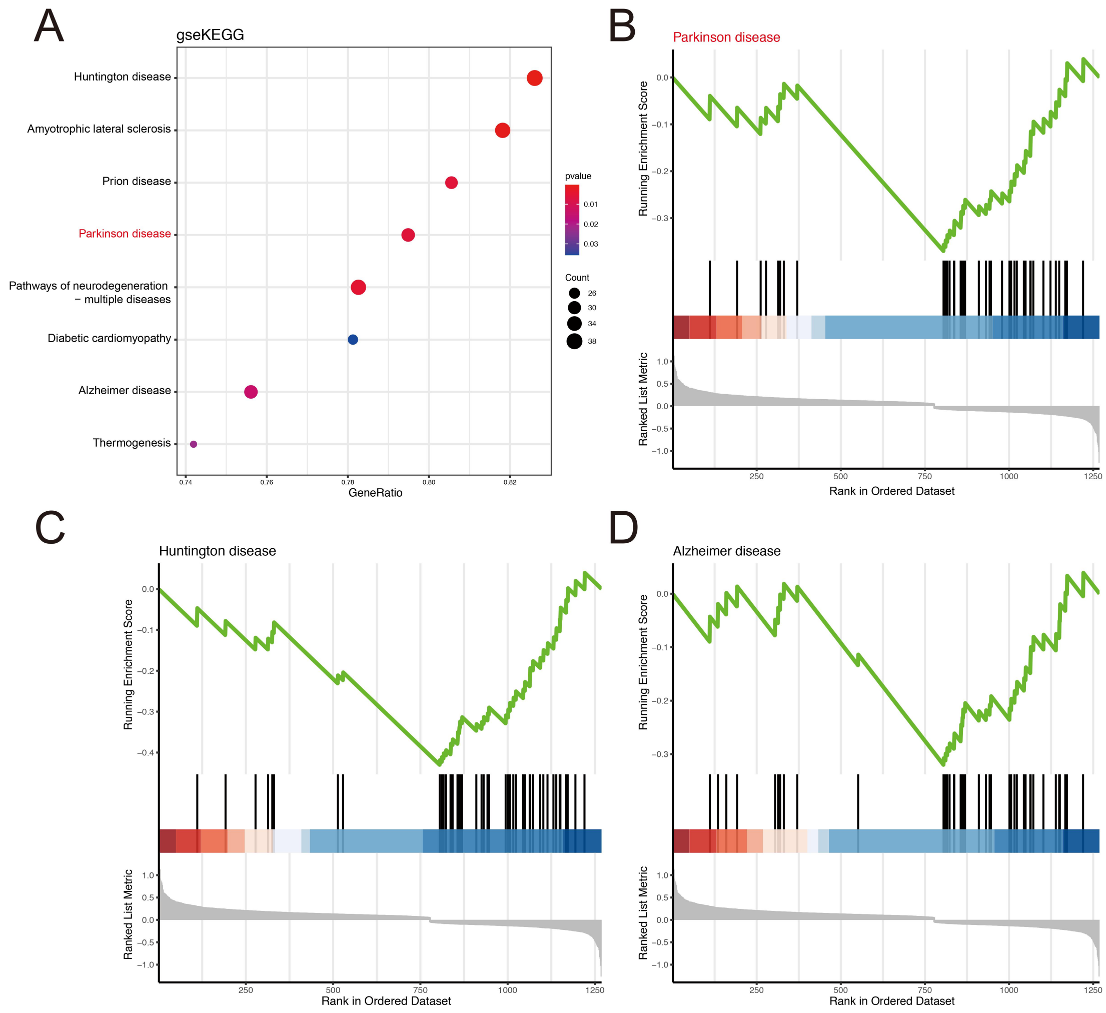

Supplementary Figure 9. GSEA based KEGG enrichment analysis of DEGs identified significantly associated with *Blautia* genus. The gseKEGG showed that DEGs associated with *Blautia* genus ($|r| > 0.3$ & $p < 0.05$) were mainly targeted to neurodegenerative diseases, including PD, HD, ALS and AD, and metabolic diseases like Diabetic cardiomyopathy (DCM) (**A**). **B, C, D** showed the graph of GSEA describing the DEGs distribution in PD (**B**) (red word), HD (**C**), and AD (**D**).
