# Supplementary material for: Integrated Microbiome and Host Transcriptome Profiles Link Parkinson’s Disease to Blautia Genus: Evidence From Feces, Blood, and Brain
Source: Front Microbiol. 2022 May 26;13:875101. doi: 10.3389/fmicb.2022.875101 (PMC9204254; doi:10.3389/fmicb.2022.875101)
Supplement: Supplementary file 20 [file Image_10.PDF]

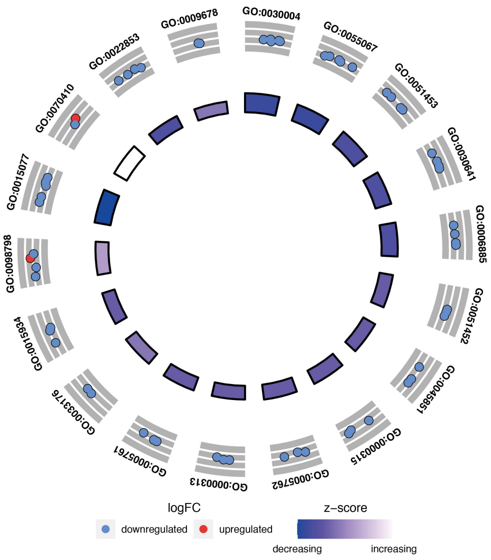

ID	Description
GO:0030004	cellular monovalent inorganic cation homeostasis
GO:0055067	monovalent inorganic cation homeostasis
GO:0051453	regulation of intracellular pH
GO:0030641	regulation of cellular pH
GO:0006885	regulation of pH
GO:0051452	intracellular pH reduction
GO:0045851	pH reduction
GO:0000315	organellar large ribosomal subunit
GO:0005762	mitochondrial large ribosomal subunit
GO:0000313	organellar ribosome
GO:0005761	mitochondrial ribosome
GO:0033176	proton−transporting V−type ATPase complex
GO:0015934	large ribosomal subunit
GO:0098798	mitochondrial protein complex
GO:0015077	monovalent inorganic cation transmembrane transporter activity
GO:0070410	co−SMAD binding
GO:0022853	active ion transmembrane transporter activity
GO:0009678	pyrophosphate hydrolysis−driven proton transmembrane transporter activity

Supplementary Figure 10. GO enrichment analysis of the overlapped 36 DEGs. There were 36 DEGs overlapped between RNA-Seq and microarray, which were significantly associated with *Blautia* genus ($|r| > 0.3$ & $p < 0.05$). The overlapped DEGs were mainly involved in pH regulation, mitochondrial function and proteasome mediated protein degradation. Only two genes increased, while all other genes decreased in the brain of PD patients.
