# Supplementary material for: Integrated Microbiome and Host Transcriptome Profiles Link Parkinson’s Disease to Blautia Genus: Evidence From Feces, Blood, and Brain
Source: Front Microbiol. 2022 May 26;13:875101. doi: 10.3389/fmicb.2022.875101 (PMC9204254; doi:10.3389/fmicb.2022.875101)
Supplement: Supplementary file 21 [file Image_11.PDF]

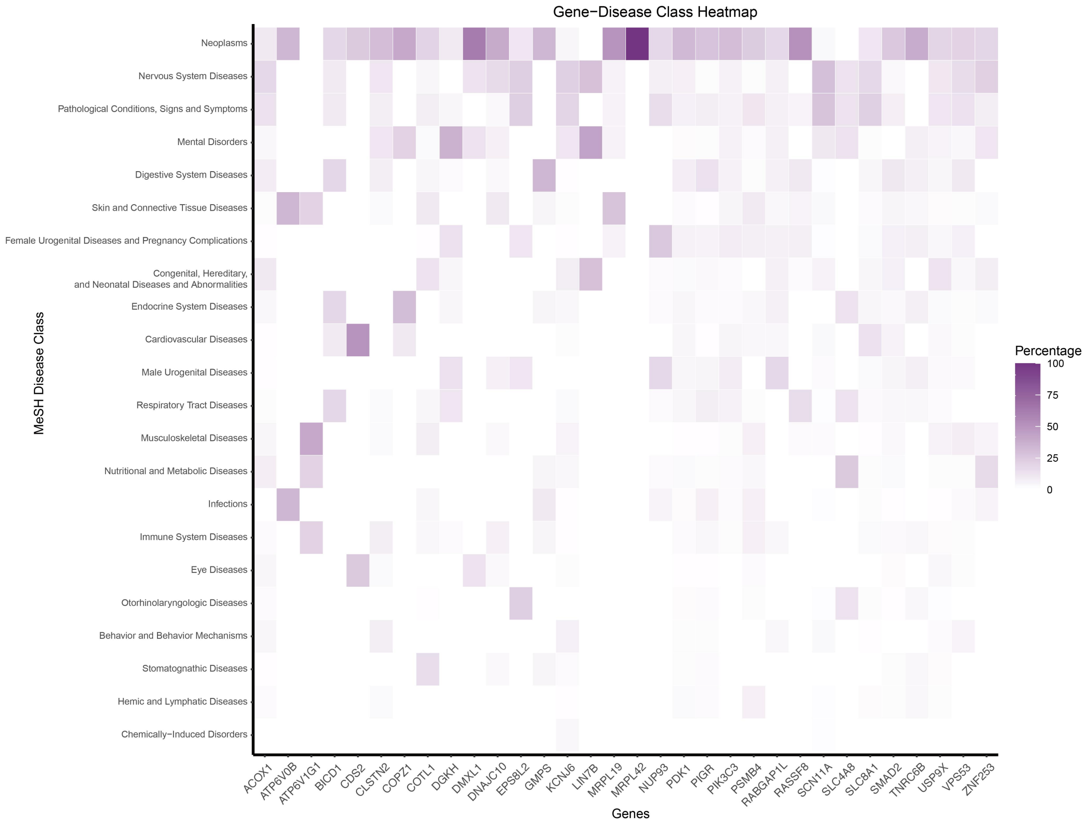


**Supplementary Figure 11. Disease class enrichment analysis of the overlapped 36 DEGs in DisGeNET database.** The overlapped 36 DEGs (|r| > 0.3 & p < 0.05) mainly participated in nervous system diseases and digestive system diseases, indicating a role of *Blautia* genus related host genes in connecting gut and brain.
