# Supplementary material for: Integrated Microbiome and Host Transcriptome Profiles Link Parkinson’s Disease to Blautia Genus: Evidence From Feces, Blood, and Brain
Source: Front Microbiol. 2022 May 26;13:875101. doi: 10.3389/fmicb.2022.875101 (PMC9204254; doi:10.3389/fmicb.2022.875101)
Supplement: Supplementary file 22 [file Image_12.PDF]

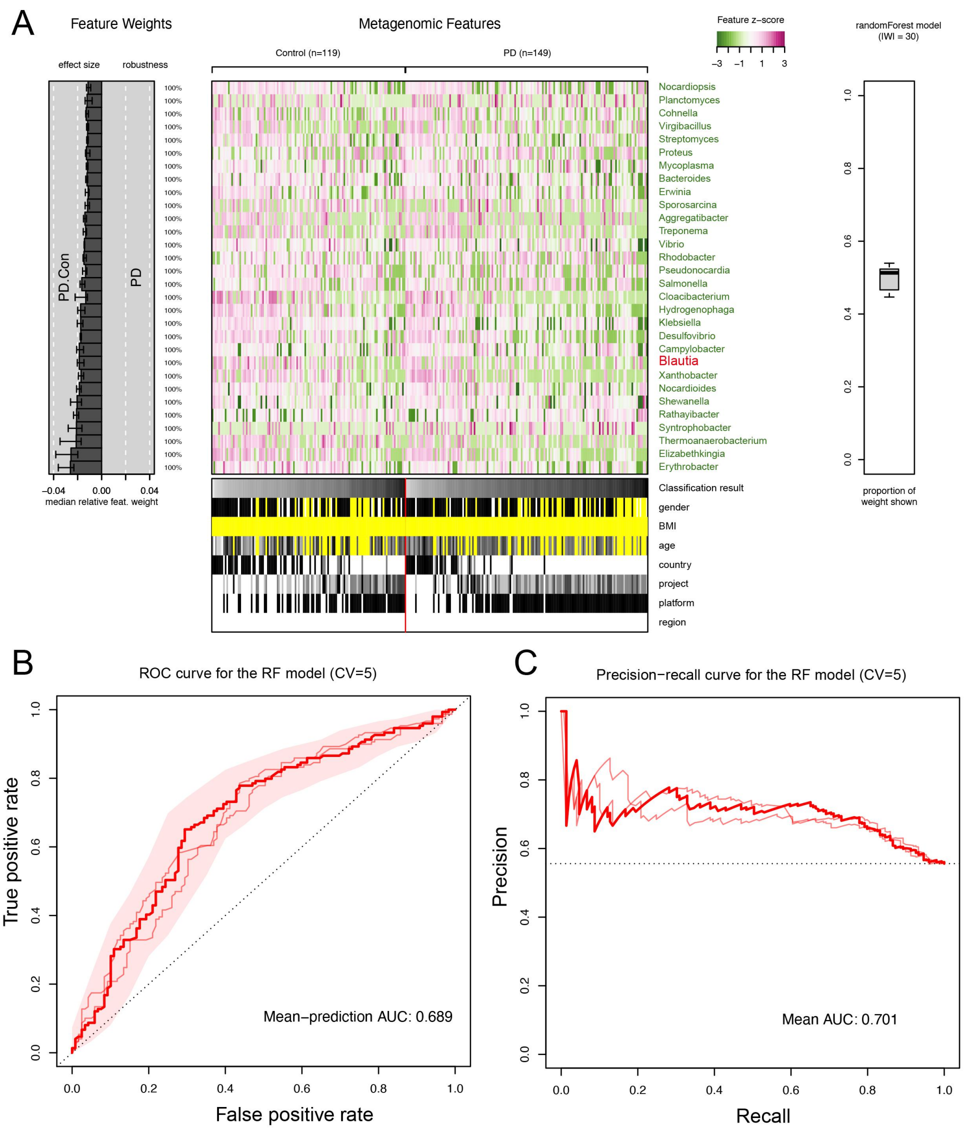

Supplementary Figure 12. Interpretation and evaluation plot of the RF model across studies for detecting PD in brain samples. The top 30 important features (genera) contributing to the predictive power of the RF model were presented in **A**, which showed a relatively good power in detecting PD. It showed that *Blautia* genus was the 9th important features for RF model in discriminating PD patients from controls, and was also decreased in the brain of PD patients as the same to data found in fecal samples. The ROC curve was plotted and AUC value of RF from five

folds cross-validation (CV, 5 folds) was 0.689 (**B**). The PRC and AUC (0.701) value was presented in **C**. The cases and controls in the RF were evenly distributed, and the AUC values from ROC and PRC were similar.
