# Supplementary material for: Integrated Microbiome and Host Transcriptome Profiles Link Parkinson’s Disease to Blautia Genus: Evidence From Feces, Blood, and Brain
Source: Front Microbiol. 2022 May 26;13:875101. doi: 10.3389/fmicb.2022.875101 (PMC9204254; doi:10.3389/fmicb.2022.875101)
Supplement: Supplementary file 23 [file Image_13.PDF]

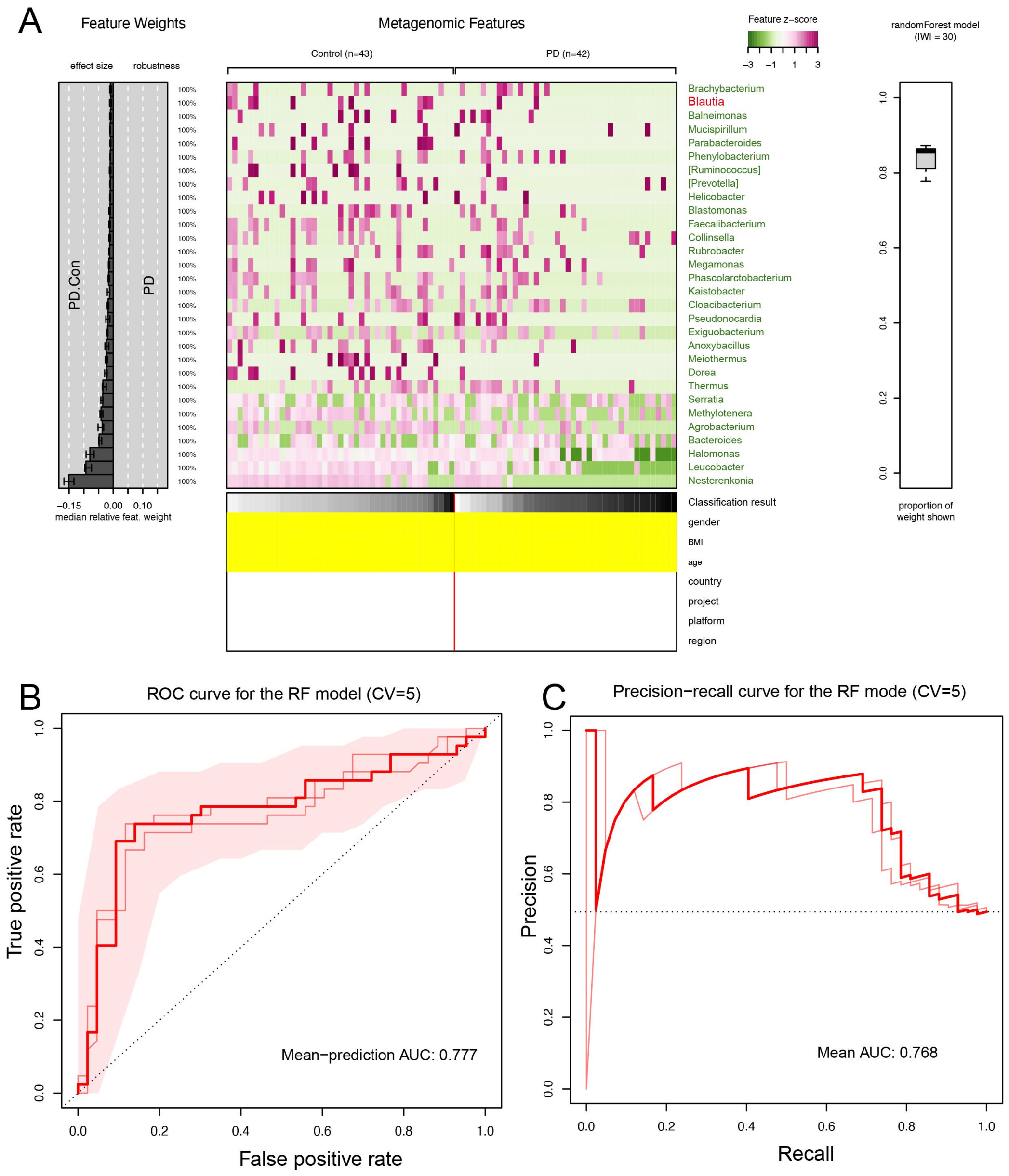

Supplementary Figure 13. Interpretation and evaluation plot of the RF model across studies for detecting PD in blood samples. The top 30 important features (genera) contributing to the predictive power of the RF model were presented in **A**. It showed that *Blautia* genus was decreased in the blood of PD patients and was one of the top 30 features (29th) for RF model. The ROC curve and AUC value (0.777) of RF from five folds cross-validation (CV, 5 folds) was showed in **B**. The PRC and

AUC (0.768) value was presented in **C**. The cases and controls in the RF were evenly distributed, and the AUC values from ROC and PRC were similar.
